# Long Intergenic Noncoding RNA MIAT as a Regulator of Human Th17 Cell Differentiation

**DOI:** 10.3389/fimmu.2022.856762

**Published:** 2022-06-15

**Authors:** Mohd Moin Khan, Meraj Hasan Khan, Ubaid Ullah Kalim, Sofia Khan, Sini Junttila, Niklas Paulin, Lingjia Kong, Omid Rasool, Laura L. Elo, Riitta Lahesmaa

**Affiliations:** ^1^ Turku Bioscience Centre, University of Turku and Åbo Akademi University, Turku, Finland; ^2^ InFLAMES Research Flagship Center , University of Turku, Turku, Finland; ^3^ Turku Doctoral Programme of Molecular Medicine, University of Turku, Turku, Finland; ^4^ The Broad Institute of Massachusetts Institute of Technology (MIT) and Harvard, Cambridge, MA, United States; ^5^ Center for Computational and Integrative Biology, Massachusetts General Hospital, Boston, MA, United States; ^6^ Institute of Biomedicine, University of Turku, Turku, Finland

**Keywords:** Th17 cells, long intergenic non-coding, MIAT, STAT3, RNA-seq, gene regulation, transcription factor

## Abstract

T helper 17 (Th17) cells protect against fungal and bacterial infections and are implicated in autoimmunity. Several long intergenic noncoding RNAs (lincRNA) are induced during Th17 differentiation, however, their contribution to Th17 differentiation is poorly understood. We aimed to characterize the function of the lincRNA Myocardial Infarction Associated Transcript (MIAT) during early human Th17 cell differentiation. We found MIAT to be upregulated early after induction of human Th17 cell differentiation along with an increase in the chromatin accessibility at the gene locus. STAT3, a key regulator of Th17 differentiation, directly bound to the MIAT promoter and induced its expression during the early stages of Th17 cell differentiation. MIAT resides in the nucleus and regulates the expression of several key Th17 genes, including IL17A, IL17F, CCR6 and CXCL13, possibly by altering the chromatin accessibility of key loci, including IL17A locus. Further, MIAT regulates the expression of protein kinase C alpha (PKCα), an upstream regulator of IL17A. A reanalysis of published single-cell RNA-seq data showed that MIAT was expressed in T cells from the synovium of RA patients. Our results demonstrate that MIAT contributes to human Th17 differentiation by upregulating several genes implicated in Th17 differentiation. High MIAT expression in T cells of RA patient synovia suggests a possible role of MIAT in Th17 mediated autoimmune pathologies.

## Highlights

• LincRNA MIAT is upregulated early on in human T cells differentiating to Th17
• STAT3 directly binds at the MIAT promoter and induces its expression in Th17 cells
• MIAT positively regulates the expression of *IL17A* and several Th17 signature genes
• MIAT is expressed in T cells of synovial biopsies from rheumatoid arthritis patients

## Introduction

T helper 17 (Th17) cells secrete IL17, IL22, and IL21 and protect against opportunistic fungal and bacterial pathogens (1). However, excessive Th17 responses have been linked to several autoimmune diseases, including psoriasis, rheumatoid arthritis (RA), and multiple sclerosis (MS) (2). Besides, Th17 cells have been linked to various types of cancers: both with a favourable, e.g., in ovarian cancer (3) or adverse prognoses, e.g., in hepatocellular and pancreatic carcinomas (4). Thus, a detailed molecular understanding of Th17 cell differentiation and function is required to target the disease-specific Th17 response and spare the non-pathogenic Th17 cell functions. Although the cytokines, signalling intermediates, and the transcriptional changes associated with Th17 cell differentiation are well known (2), little is known about the contribution of long intergenic noncoding RNA (lincRNA) in human Th17 cell differentiation.

LincRNAs are long (>200 bp), independently transcribed, non-coding RNAs. They participate in normal cellular functions and disease development by regulating transcriptional and post transcriptional gene expression. Their key functions include cellular signaling, gene expression, and chromatin remodeling (5). We and others identified several lincRNAs that are differentially regulated during Th17 differentiation in humans and mice (6, 7). However, the roles of individual lincRNAs in human Th17 cell differentiation are poorly understood.

The lincRNA Myocardial Infarction Associated Transcript (MIAT) is associated with a genetic susceptibility towards myocardial infarction (MI). A genome-wide case-control study of single nucleotide polymorphisms (SNP) showed six SNPs near MIAT to be strongly associated with MI. One of those, rs2301523, altered MIAT expression in *in-vitro* assays (8). Enhanced MIAT expression has also been reported in lymphocytic leukemia (9) and diabetes mellitus (10). Recent studies also suggest a role of MIAT in Th17 differentiation in mice (11, 12).

Here, we studied the regulation of MIAT expression during human Th17 differentiation. We also showed that MIAT regulates the expression of IL17A and other Th17 related genes during human Th17 cell differentiation by altering the chromatin accessibility of key loci. Interestingly, MIAT was highly expressed in T cells of RA patient synovial biopsies and psoriatic skin lesions, suggesting a role in autoimmune diseases.

## Materials and Methods

### Human CD4^+^ T-Cell Isolation, Activation, and Differentiation

CD4^+^ T cells were isolated from human umbilical cord blood using bead-based positive selection. The cells were differentiated to Th1 or Th2 (13), Th17 (7) or iTreg (14) subsets as described earlier.

For Th17 polarization, cells were activated using a plate coated anti-CD3 antibody (Beckman Coulter, cat# IM-1304) (3750 ng/well in a six-well culture plate), and anti-CD28 antibody (Beckman Coulter, cat# IM1376) was added in the medium in a concentration of 1 μg/mL. X-vivo 20 serum-free medium (Lonza, Bazel, Switzerland) containing 0.5 million cells/mL complemented with L-glutamine (2 mM, Sigma-Aldrich) and antibiotics (50 U/mL penicillin and 50 μg/mL streptomycin; Sigma-Aldrich) was used for the cultures. Cells were plated at a density of 2 million cells per well and cultured at 37°C in 5% CO2. IL-6 (20 ng/mL; Roche, cat# 11138600 001), IL-1β (10 ng/mL, R&D Systems, cat# 201 LB) and TGFβ (10 ng/mL, R&D Systems, cat# 240) were used as the cytokine cocktail along with neutralizing antibodies against IFNγ (1 μg/mL, R&D Systems, cat# MAB-285) and IL4 (1 μg/mL, R&D Systems, cat# MAB204) in the Th17 cultures. Nonpolarizing control (Th0) cells were activated with anti-CD3 and anti-CD28 antibodies in the presence of the neutralizing antibodies without differentiating cytokines. Number of donors varied from experiment to experiment, but it was never less than three independent biological replicates, either individual cord blood samples or from the pool of 3-5 individuals.

### Cell Transfections

150 pmols of Locked Nucleic Acid Antisense Oligonucleotides (LNAs) were transfected into 4 million cells in 100 μL volume of OptiMEM (Gibco by Life Technologies, cat # 31985-047), using the Amaxa Nucleofector II system (Lonza). For siRNA transfections, 6.6 μg of PRKCA SMARTpool siRNA from Dharmacon (Cat# M-003523-03-0005) was used. After transfection, cells were first rested at 37°C for 48 h (LNA) or 24h (siRNA) in RPMI 1640 (Sigma-Aldrich) medium supplemented with 10% FCS, 50 U/mL penicillin, 50 μg/mL streptomycin, and 2 mM L-glutamine and then activated and differentiated. LNA and siRNA sequences are shown in **Supplementary Methods**. The transfection details for PKCα rescue experiments has been described below in the section titled “Generating PRKCA *in-vitro* transcribed (IVT) RNA and its overexpression”.

### Flow Cytometry

Flow cytometry analysis of cell-surface receptor CCR6 detection (using anti-CCR6, cat# 559562, BD Bioscience) was performed at 72 h as described earlier (15). To measure PKCα overexpression at protein level, FACS samples were collected six hours after *in vitro* transcribed PKCa RNA had been introduced to cells by nucleofection. The cells were fixed using pre-warmed BD Phosflow Fix buffer I (BD, Cat. no. 557870) for 10 minutes at 37°C. The cells were collected after centrifugation and resuspended in 300ul of cold Perm buffer III and incubated at +4°C for 30 minutes. Perm buffer was removed and cells were washed in 200ul of Stain buffer (BD Cat#554656). The PKCα antibody (Cell signaling Cat# 59754) was used in 1:100 dilution in staining buffer and the samples were incubated at room temperature for 30 minutes. Cells were washed two times and resuspend in staining buffer containing Alexa fluor-647 secondary antibody (cell signaling Cat no. 4414) for 30 minutes at room temperature. Cells were washed for two times and resuspend in PBS for FACS analysis. The data was acquired in BD LSR II/LSRFortessa. The acquired data were then analyzed either by Flowjo (FlowJo LLC) or Flowing software (https://bioscience.fi/services/cell-imaging/flowing-software/).

### TaqMan Quantitative Real-Time-PCR

RNA was isolated using RNeasy^®^ Mini Kit (Qiagen, cat #74106) following manufacturer’s instruction followed by cDNA synthesis and TaqMan analysis as we described earlier (15). The primers are listed in **Supplementary Methods**. In all TaqMan gene expression assays, the Ct values were normalized to the expression of the house keeping gene EEF1A1 (16).

### Western Blotting

Western blotting was performed as descried earlier (15). Briefly, cells were lysed in Triton-X-100 lysis buffer. The lysates were resolved on the acrylamide gel and transferred to PVDF membrane, which were incubated with primary and secondary antibodies in 5% BSA in tris buffered saline. The following antibodies were used: PKCα (Cell Signaling: cat# 59754), and beta-actin (Sigma, cat# A5441). The western blotting bands were quantified to measure the relative protein concentration using imageJ software (https://imagej.nih.gov/ij/docs/menus/analyze.html) and normalized to β-Actin, which was used as a loading control.

### RNA Sequencing (RNA-seq)

RNA-seq Illumina TruSeq^®^ Stranded library preparation was performed following Illumina protocols (part # 15031047). The libraries were sequenced at the Finnish Functional Genomics Centre using the HiSeq2500. Data quality check, trimming, alignment, read count and differential expression was performed using FastQC (17), Trimmomatic (18), Tophat2 (19), HTSeq (20), and DEseq (21), respectively. For more details, please see **Supplementary Methods**. Genes with a false discovery rate (FDR) <0.05 were considered significant.

### Assay for Transposase-Accessible Chromatin Using Sequencing Analysis (ATAC-seq)

MIAT deficient and sufficient cells were differentiated under Th17 conditions for 72 h in three biological replicates. Nuclei were isolated, and the libraries were prepared as described earlier (22). Nextera DNA library preparation kit with i7 and i5 indices (15028212) and AMPURE-XP beads (B46053, Beckman Coulter) were used to purify the libraries. Read quality-check and adapter trimming was performed using FastQC (v.0.11.4) (17) and TrimGalore (v. 0.4.5) (https://www.bioinformatics.babraham.ac.uk/projects/trim_galore/), respectively. The trimmed reads were mapped to the hg38 reference genome using Bowtie2 (v. 2.3.3.1) (23). Open chromatin regions were identified and quantified using the peak caller MACS2 (v. 2.1.0) (24). MACS2 quantifies the peak signal by calculating a fold enrichment value for the peak summit against random Poisson distribution with local lambda, which is a dynamic parameter defined for each candidate peak. Local lambda captures the influence of local biases and is robust against occasional low read counts at small local regions. The regions were annotated using HOMER (v.4.9) (25).

### ATAC-qPCR

For PCRs of *IL17A* locus, the primers were designed targeting the three regions at *IL17A* promoter within the highlighted region in **Figure 3F**. The TaqMan qPCR was performed on PCR amplified libraries using the primers listed in **Supplementary Methods**.

### Luciferase Reporter Assay

CD4^+^ cells were polarized to Th2 and Th17 cells for 3 days and transiently nucleofected with a promoter-luciferase reporter plasmid. After 48 h resting the cells were reactivated under Th2 and Th17 conditions for 24 h, harvested in passive lysate buffer (provided in the Dual-Luciferase Reporter Assay from Promega, cat# E1910) and luciferase activity was measured according to the manufacturer’s instructions. GenScript commercially synthesized the plasmids of the luciferase reporter assay. In each sample, firefly luciferase values were normalized to renilla luciferase values and plotted as a fold change over empty luciferase construct pGL4-minP. To mutate the STAT3 binding site, following changes were made at all places within the luciferase construct: GGAA was replaced with CCCC, TTCC was replaced with AAAA and TTCCAGTAA replaced with AAAAAAAAA.

### Chromatin Immunoprecipitation qPCR (ChIP)-qPCR

**Figure 1:**
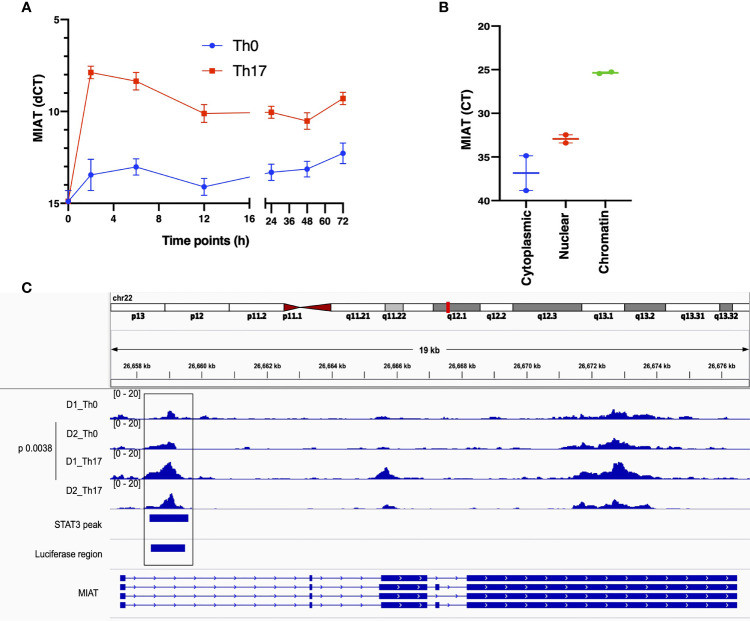
MIAT is Rapidly Induced in Human Th17 and Th0 Cells and Primarily Localized in the Nucleus. **(A)** The line plot shows MIAT expression in Th17 and Th0 cells, as analyzed by TaqMan qPCR analysis. The CD4^+^ T cells were cultured for 0, 2, 6, 12, 24, 48, and 72 h, under Th17 and Th0 condition, and MIAT expression was detected by TaqMan qPCR. Mean +/- SEM of three independent experiments are plotted. **(B)** TaqMan qPCR analysis of MIAT localization in subcellular fractions of differentiated Th17 cells (72 h). The cytoplasmic, nuclear, and chromatin fractions of Th17 cells were first isolated, and MIAT expression was detected by TaqMan qPCR. Threshold cycle (Ct) denotes the number of PCR amplification cycles that were required before the expression could be detected. Lower Ct corresponds to higher expression. For both A and B, the experiments were performed using CD4^+^ T cells isolated from umbilical cord blood samples. Each experiment was repeated for 3 times, each of which using a pool of CD4^+^ T cells collected from umbilical cord blood of 3-5 individuals. **(C)** MIAT locus is visualized on the IGV genome browser (hg38). The ATAC-seq peaks of differentiating Th17 cells (72h) and their respective nonpolarizing (Th0) controls are shown. The data is obtained from the CD4^+^ T cells purified from umbilical cord blood samples collected from two donors (D1 and D2). ChIP-seq data of STAT3 binding on MIAT locus in differentiating Th17 cells (72 h) (15) and the region targeted in the luciferase assay are also shown in the bottom of the figure. The p-value denotes the statistical significance of Th0-Th17 comparison. *in situ*.

Cells were activated under Th0 and Th17 condition for 30 minutes. STAT3 ChIP was performed as described earlier (15) using STAT3 antibody (cat# 9132L, Cell Signaling Technology) and control IgG antibody. 10 µg of each antibody, was incubated with 200 µg of cell chromatin (fragments: 100–500 bp), prepared using a Bioruptor sonicator (Diagenode), and crosslinked with magnetic beads (no. 112.04 Dynal Biotech, Invitrogen). The cross-linking was reversed at 65°C for 12h and DNA was precipitated by Proteinase K and RNase A and purified by QIAquick PCR purification kit (QIAGEN). Immunoprecipitated DNA was used to perform real-time qPCR (7900HT Fast Real-Time PCR System, Applied Biosystems) and analyzed as described previously (15). The ChIP primers were designed to detect the STAT3 peak at MIAT promoter based on our earlier STAT3 ChIP-seq data (15) (**Figure 1C**). The primers are listed in **Supplementary Methods**.

### IL-17 Detection by ELISA or LUMINEX Assay

IL-17A levels were measured in culture supernatants as described earlier (15). One of the following kits were used: The Milliplex MAP human IL-17A kit (Merck Millipore; HCYTOMAG-60K-01); Bioplex Human IL-17A Cytokine/Chemokine 96-Well Plate Assay (Bio-Rad,cat# 171B5014M, 171304090M) or Human IL-17A Duoset ELISA kit (R&D Biosystems, cat# DY317-05, DY008). The concentration was normalized to the cell count.

### Generating PRKCA *In-Vitro* Transcribed (IVT) RNA and its Overexpression

*In vitro* RNA for GFP and PRKCA were transcribed from respective plasmids using the T7 mScript Standard mRNA Production System (CELLSCRIPT, cat# C-MSC100265) following the manufacturer’s instructions. An Agilent Bioanalyzer or BioRad Experion was used to confirm the size of the RNA. The RNA was then Capped and polyadenylated following instructions from the T7 mScript kit. Cells were first transfected with either MIAT-LNA1 or NC-LNA and cultured under Th17 condition as described above for 48 h. Cells were re-transfected with 28 picomoles of IVT generated PRKCA or GFP (used as negative control) RNA or none (mock transfection). The cells were allowed to rest for 24 h in RPMI 1640 medium supplemented with 10% FCS, 50 U/mL penicillin, 50 μg/mL streptomycin, 2 mM L-glutamine, and 17ng/ml IL2 (Cat# 101-IL; R&D systems). Cells were then activated in Th17 polarizing media for 72 h and the cell culture supernatant was collected for IL-17A ELISA. After six hours of re-transfection with IVT RNA, the overexpression was checked by FACS (**Figure S7A**).

### Pathway Analysis

Ingenuity Pathway Analysis (IPA) (https://www.ingenuity.com/) was used for pathway analysis with P-values < 0.01 to determine the statistically significant enriched pathways.

### Transcription Factor Binding Sites Motif Enrichment Analysis

MIAT silenced gene promoters (-1000 to +100 bp from TSS) were analyzed by the FMatch tool of the TRANSFAC database (Release 2019.3). We generated a custom profile of TFs expressed in T cells, and their enrichment was tested in the MIAT target gene promoters compared to a randomly generated background set of promoters. A P-value of 0.01 was considered significant.

### Statistics and Plotting

One/two-tailed paired Student’s T-test was used to find the statistical significance of the difference between means, and p-value <0.05 was considered significant. For plotting, GraphPad Prism, or R were used.

### RNAscope *In Situ* Hybridization

RNAScope experiments were performed using RNAScope multiplex fluorescent reagent kit v2 ACD a biotechne brand (cat# 323100), following manufacturer’s recommendations. More detailes are provided in **Supplementary Methods**.

### Reanalysis of Published Single-Cell RNA-Seq Data

We examined MIAT expression in immune cells from synovial tissue samples of RA patient from two studies (26, 27). Details of the analysis is provided in the **Supplementary Methods**.

## Results

### MIAT Is Induced in Human Th17 Cells and Primarily Localized in the Nucleus

During transcriptome analysis of human and mouse Th17 cells, we earlier observed MIAT upregulation in human but not in mouse Th17 cells (7). This finding prompted us to investigate the function of MIAT in human Th17 cells. First, we confirmed the Th17-specific upregulation of MIAT in independent experiments with TaqMan qPCR (**Figure 1A**). Interestingly, MIAT was rapidly and highly upregulated in T cells induced to differentiate into Th17 cells already at 2 h post activation, suggesting that MIAT is important in the initial priming of human Th17 cell differentiation. Interestingly, the expression was unchanged during early differentiation of Th1 cells and downregulated during early differentiation of Th2 and iTreg cells (**Figures S1A, B**). RNAScope *in situ* hybridization (RNA-ISH) showed MIAT to be primarily localized in the nuclear fraction in Th17 cells (**Figures S1C, D**), which was further confirmed by TaqMan analysis (**Figure 1B**).

### MIAT Locus Is Open in Human Th17 Cells

To study the epigenetic landscape of the MIAT locus in developing Th17 cells, we examined chromatin accessibility (ATAC-seq) at the MIAT locus (unpublished data). We found that the MIAT promoter was more accessible in developing Th17 cells early on than in T cells activated but not induced to differentiate (Th0) (**Figure 1C**). These results indicate that the MIAT locus is epigenetically regulated and transcriptionally active during early Th17 differentiation.

### MIAT Upregulation in Th17 Cells Is STAT3 Dependent

**Figure 2:**
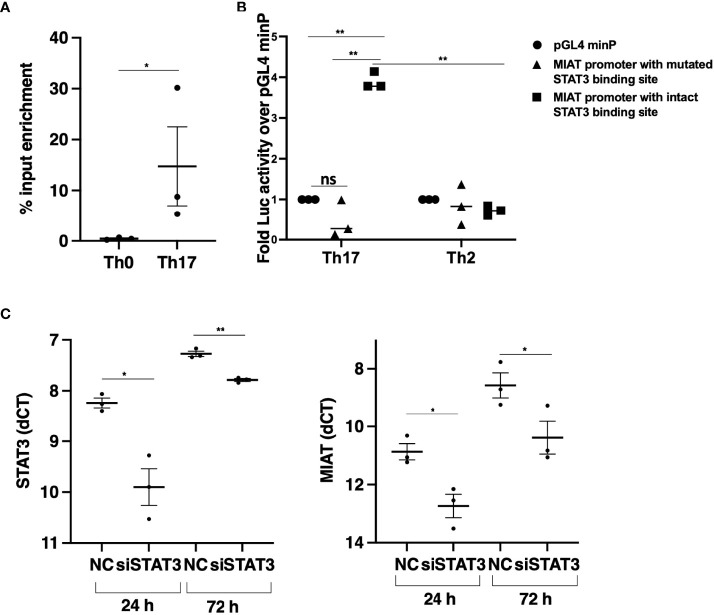
STAT3 Binds at MIAT Promoter and Upregulates its Expression in Th17 Cells. **(A)** TaqMan-qPCR of MIAT promoter performed on STAT3 ChIP of 0.5-h Th17 and Th0 cells. The percent input was used to calculate the enrichment, and error bars represent SEM. *, p < 0.05. **(B)** The promoter region (–1500 to +250 base-pair relative to TSS) was cloned upstream of the firefly luciferase coding region. The vector containing the MIAT promoter region or empty vector (pGL4 minP) was nucleofected into Th17 or Th2 cells, and the firefly luciferase activity was measured. The data were normalized to renilla luciferase for transfection efficiency and presented with respect to the control vector. For details, see the method section and **Figure S2B** for schematics. Error bars represent SEM of three experiments. Statistical analysis was performed by paired two-tailed Student’s t-test. *, *p* < 0.05. **(C)** STAT3 (left) and MIAT (right) expression in STAT3-silenced Th17 cells by TaqMan qPCR analysis. The STAT3 siRNAs (siSTAT3) were used to knockdown STAT3 in three individual donors before Th17 polarization for 24 and 72 h. Non-targeting control siRNA (siNC) was used as control. Each dot represents an individual donor. Students’ paired two-tailed t-test was used to determine the significance. *p <0.05; **p <0.01. In all the panels, the experiments were performed on three pools of CD4^+^ T cells, where each pool contained cells purified from umbilical cord blood collected from 3-5 individuals.

Next, we analyzed the promoter of MIAT to study the transcription factor binding sites (TFBS) for TFs that drive Th17 cell differentiation. We showed earlier that STAT3 phosphorylation occurs within 30 min after IL-6R stimulation upon induction of Th17 differentiation (15). Further, based on a genome-wide analysis of STAT3 binding in the same study, we found a STAT3 binding site at the MIAT promoter (**Figure S2A**) (15). Thus, we hypothesized that STAT3 binds to the MIAT promoter and regulates its transcription. We confirmed STAT3 binding on MIAT promoter with chromatin immunoprecipitation (ChIP) and real-time qPCR analysis (**Figure 2A**). To determine if the binding of STAT3 is functional, we used a luciferase assay (**Figure S2B**). The construct containing the promoter region of MIAT, including the STAT3 binding site, -1500 to + 250 from MIAT transcription start site (TSS), was cloned next to luciferase reporter gene and transfected to Th17 cells (72 h after initiation of differentiation), where STAT3 is highly expressed and phosphorylated (15). Th2 polarized (72 h) cells, where STAT3 is not in an active form, served as controls, as reported (28). Higher luciferase activity was detected in Th17 cells transfected with constructs with MIAT promoter containing STAT3 binding site than in Th17 cells transfected with control constructs (**Figure 2B**). No luciferase activity was seen in Th2 cells or cells transfected with construct where STAT3 biniding site was mutated (**Figure 2B**). Thus, STAT3 binding on the MIAT promoter resulted in transcriptional activity. Further, the transfection of the construct where STAT3 binding site was mutated did not lead to any increase in luciferase activity. Furthermore, we confirmed the regulation of MIAT by STAT3 with RNA interference. STAT3 silencing in 24 h- and 72 h- polarized Th17 cells led to reduced MIAT expression (**Figure 2C**).

### MIAT Positively Regulates Human Th17 Differentiation

**Figure 3:**
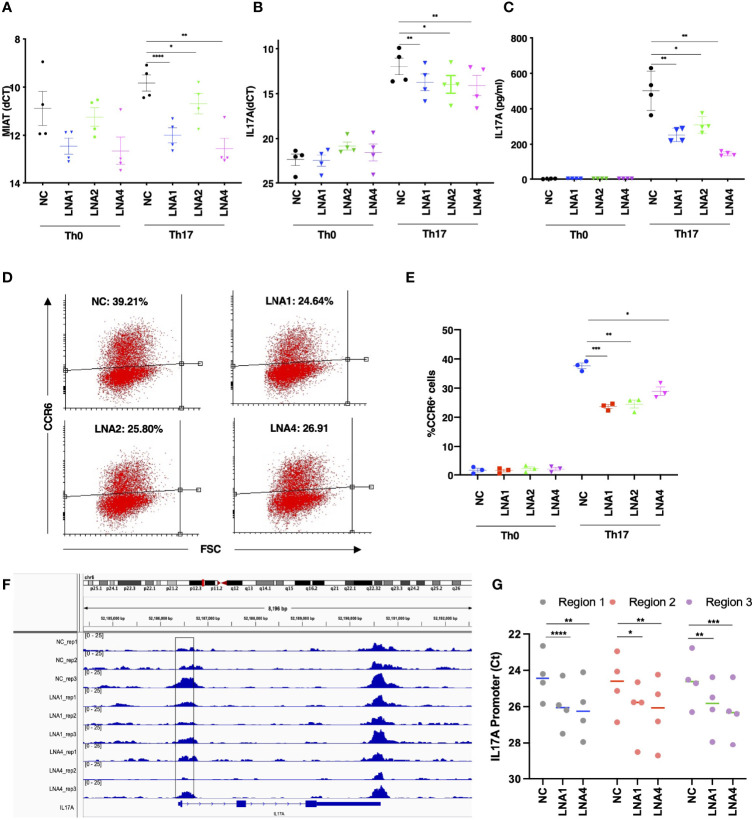
MIAT Positively Regulates Human Th17 Cell Differentiation. **(A)** TaqMan qPCR analysis of MIAT-silenced Th0 and Th17 (72 h) cells. Three different LNAs (LNA1, LNA2, and LNA4), targeting different MIAT regions, were used to silence MIAT. NC represents non-targeting LNA used as a negative control. Each dot represents an independent experiment perfomed on T cells purified from umbilical cord blood samples collected from different individuals. Error bars represent SEM. Students’ paired two-tailed t-test was used to determine the significance. *, p <0.05; **, p <0.01; ****, p <0.0001. **(B, C)** The TaqMan qPCR analysis of *IL17A* expression **(B)** and IL-17A secretion detected by Luminex assay **(C)** in MIAT silenced, 72 h polarized Th17, and Th0 cells. Students’ paired two-tailed t-test was used to determine the significance. *, p <0.05; **, p <0.01. In panels A-C, the experiments were performed on four pools of CD4^+^ T cells, where each pool contained cells purified from umbilical cord blood collected from 3-5 individuals. **(D, E)** Flow cytometry analysis of CCR6 expression in MIAT-silenced control (Th0) and Th17 polarized cells (72 h). Panel **(D)** shows the scatter plots from one experiment, and **(E)** shows the data from all three experiments. The experiments were performed on three pools of CD4^+^ T cells, where each pool contained cells purified from umbilical cord blood collected from 3-5 individuals. Error bars represent SEM. Students’ paired two-tailed t-test was used to determine the significance. *, p <0.05; **, p <0.01; ***, p <0.001. **(F)** Visualization of ATAC-seq data at IL17A locus. Data from three biological replicates are shown. The highlighted region was validated by ATAC-PCR. The experiments were performed on three pools of CD4^+^ T cells, where each pool contained cells purified from umbilical cord blood collected from 3-5 individuals. **(G)** ATAC-seq library was amplified using primers to detect the three subregions of the highlighted regions in panel **(F)** The experiments were performed on CD4^+^ T cells, from umbilical cord blood collected from 4 individuals. Statistical significance was determined using paired two tailed t-test. *, p <0.05; **, p <0.01; ***, p <0.001, ****<0.0001.

To study the influence of MIAT on human Th17 differentiation, we silenced MIAT in Th17 cells. Three LNAs targeting different regions of the MIAT sequence were used along with non-targeting control (NC) LNA. MIAT depletion (**Figure 3A**) resulted in reduced IL-17A expression at the RNA and protein levels (**Figures 3B, C**). Further, we also measured CCR6 surafce expression as a marker indicating Th17 differentiation, and found it to be significantly downregulated in the MIAT-deficient cells (**Figures 3D, E**, **Figure S3**). Interstingly, MIAT silencing did not alter the expression of CD69 (**Figure S4A**) or RORC (**Figure S4B**) on Th17 cells. Further, MIAT silencing did not significantly influence the expression of genes encoding Th1 cell- or Th2 cell-specific transcription factors *TBX21* or *GATA3* or *cytokines IFNG*, and *IL4* (**Figures S5A-E**). Differentiation to Th1 and Th2 cells was not compromised. Comparing LNA NC treated Th0 and Th1 cells, there was over 2 fold difference in TBX21 expression (**Figure S5B**). Similarly, comparing LNA NC treated Th0 and Th2 cells, there was over 2 fold difference in GATA3 expression (**Figure S5D**).

Next, we analyzed the genome-wide chromatin accessibility to identify open and closed chromatin by ATAC-seq in MIAT silenced Th17 cells. Twenty four and 57 loci were differentially accessbile upon MIAT silencing by LNA1 at 24 and 72 h, repectively. Relatively larger number of loci, 182 and 409 were differentially accessibile upon MIAT silencing by LNA4 at 24 and 72 h, respectively (**Table S1**). Interestingly, we observed that IL17A promoter was less accessible in MIAT silenced Th17 cells compared to controls (**Figure 3F**). Based on the ATAC-seq analysis in MIAT silenced and control Th17 cells, three different regions of *IL17A* promoter were selected for validations by ATAC-seq TaqMan qPCR analysis (highlighted **Figure 3F**). Indeed, reduced chromatin accessibility of IL17A locus upon MIAT depletion was confirmed in four replicates (**Figure 3G**). We visualized the effect of MIAT silencing on the accessibility of other Th17 related loci, and found that loci near CXCL13 and RORA genes had less accessible regions, particularly upon LNA4 treatment (**Figures S6A, B**). However, no significant effect could be observed at other Th17 loci, e.g., RORC.

Further, we studied the TFBS on the differentially accessibile regions and found enrichment of 54 TFBS to be common in the genomic regions differentially accessbile upon treatment with LNAs at different time points (**Figure S6C**). Besides JUN and FOS TFs, the list includes several known Th17 associated factors, e.g., FOSL1, FOSL2, BATF and MAF (**Table S1**).

### MIAT Controls Th17 Differentiation by Regulating Expression of PKC-α

**Figure 4:**
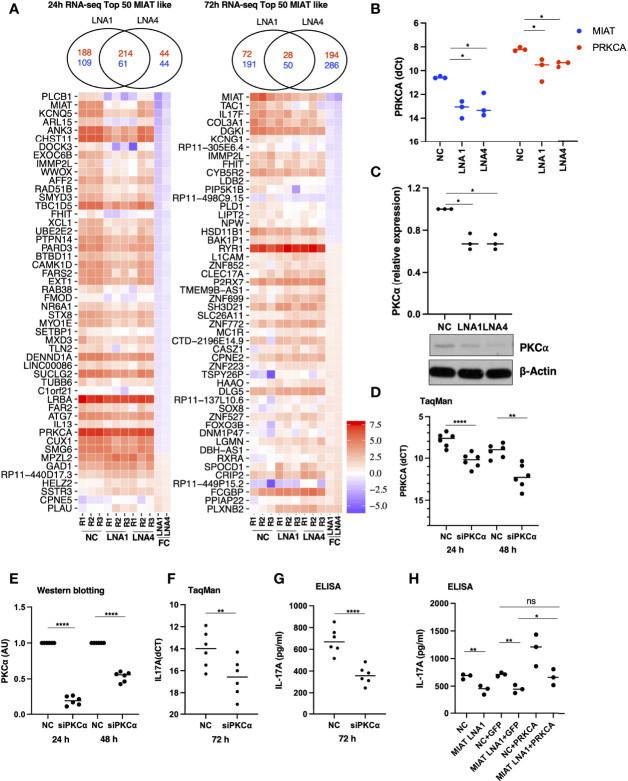
RNA-seq Analysis Identified MIAT Targets in Human Th17 Cells. **(A)** Venn diagrams on the top show the overlap of the number of differentially regulated genes (FDR <0.05 and LFC >1.5) when comparing LNA1- or LNA4-transfected samples to the negative control LNA (NC). The numbers in red indicate common and unique upregulated genes, and numbers in blue indicate common and unique downregulated genes upon MIAT silencing. Heatmap representation of the expression of the top 50 DE genes similarly regulated (see results section for details) upon MIAT silencing at 24 (left) and 72 h (right) in Th17 cells. NC, LNA1 and LNA4 columns represent the gene expression (RPKM) in respective samples. FC columns represent the fold-change in gene expression upon MIAT silencing. **(B)** TaqMan qPCR of PRKCA expression analysis in MIAT silenced Th17 cells with two different LNAs at 24 h, where each dot represents an independent experiment. Students’ paired two-tailed t-test was used to determine the significance. *, p <0.05. **(C)** Western blot expression analysis of PKCα proteins in MIAT-silenced 24-h Th17 cells. A representative result (lower panel) from three independent experiments for PKCα is shown along with β-actin for loading control. WB quantification was performed using ImageJ, from three independent experiments (upper panel), and significance was calculated using Students’ t-test (two-tailed, paired). *, p <0.01. The data were plotted as arbitrary units (AU). **(A-C)** The experiments were performed on three pools of CD4^+^ T cells, where each pool contained cells purified from umbilical cord blood collected from 3-5 individuals. **(D)** TaqMan qPCR of PRKCA expression analysis showing knockdown efficiency of the siRNA at RNA level at 24 h and 72 h. Students’ paired two-tailed t-test was used to determine the significance. **, p <0.01; ****, p <0.0001. **(E)** Western blot expression analysis of PKCα proteins showing knockdown efficiency of the siRNA at protein level at 24 h and 72 h The quantification was performed using ImageJ, from three independent experiments, each having two biological replicates. The intensities were normalized to β-actin. The significance was calculated using Students’ t-test (two-tailed, paired). ****, p <0.0001. The data were plotted as arbitrary units (AU). **(F)** TaqMan qPCR of IL17A expression from three independent experiments, each having two biological replicates. The Ct values were normalized to EF1A. The significance was calculated using Students’ t-test (two-tailed, paired). **, p <0.01. **(G)** IL-17A secretion as measured by ELISA. The data was obtained from three independent experiments, each having two biological replicates. The data has been normalized to number of cells in the culture-well. The significance was calculated using Students’ t-test (two-tailed, unpaired). ****, p <0.0001. **(D-G)** The experiments were performed on six pools of CD4^+^ T cells, where each pool contained cells purified from umbilical cord blood collected from 3-5 individuals. **(H)** IL-17A secretion as measured by ELISA. The scheme for this experiment has been shown in **Figure S7A**. In the first nucleofection cells were transfected with NC or MIAT targeting LNA, whereas in the second nucleofection the cells were either mock transfected or they were transfected with *in vitro* transcribed GFP or PRKCA RNA. The experiments were performed on three pools of CD4^+^ T cells, where each pool contained cells purified from umbilical cord blood collected from 3-5 individuals. The significance was calculated using Students’ t-test (two-tailed, paired). **, p <0.01; *, p <0.05; ns denotes not significant.

To identify the transcriptional targets of MIAT in human Th17 cell differentiation, we used genome-wide gene expression profiling of MIAT-deficient Th17 cells. We performed RNA-seq in three biological replicates of MIAT-silenced Th17 cells at 24 and 72 h. Two LNAs targeting different regions of MIAT were used in the RNA-seq experiments. In total, 572 and 363 genes were differentially expressed (DE) (LFC 0.59; FDR 0.05) at 24 h (**Table S2**), whereas 341 and 557 genes were DE at 72 h in LNA1- and LNA4-treated samples, respectively (**Table S2** **;** **Figure 4A**). MIAT was among the most downregulated genes at both time points, confirming efficient knockdown (**Figure 4A**). IL17F was significantly downregulated in MIAT-silenced cells at 72 h (**Figure 4A**). IL17A was also downregulated more than two-fold at 72 h, though, the p-values were just above the significance threshold (LNA1: LFC -1.047, FDR 0.059; LNA4: LFC -1.050, FDR 0.051) (**Table S2**).

For further analysis, we only took those genes as MIAT targets that were DE by both LNAs with the same direction of change: 275 genes for 24 h and 78 genes for 72 h (**Figure 4A**). 345 MIAT target genes (union of 275 and 78 genes) included 15 lincRNAs, of which three (MIAT, ERVH48-1, RP11-18H21.1) were also DE during Th17 differentiation in our earlier timeseries RNA-seq analysis (7). Interestingly, RP11-18H21.1 (also known as LINC02273) was down-regulated during Th17 differentiation (7) but upregulated upon MIAT silencing.

The rapid induction of MIAT expression during Th17 differentiation suggested that it regulates early events in the process. PKC-α (encoded by *PRKCA*) is among the early signaling molecules activated in response to T-cell activation. It regulates human Th17 cell differentiation by modulating TGF-β signaling through SMADs (29). In the RNA-seq data, PRKCA was downregulated upon MIAT silencing using both LNAs at 24 h. We confirmed the downregulation of PKCα in MIAT-silenced Th17 cells by western blot (WB) and TaqMan PCR analysis (**Figures 4B, C**). These data suggested that MIAT may regulate IL17A expression through PKCα.

To test if PKCα indeed regulates IL17A expression in human Th17 cells, we silenced PKCα expression using siRNA (**Figures 4D, E**). Interestingly, silencing PKCα resulted in reduced IL17A expression at the RNA level and its secretion in the culture supernatant (**Figures 4F, G**). To test our hypothesis that the effect of MIAT on IL17A could be mediated through PKCα, we overexpressed PKCα in MIAT deficient Th17 cells. The overexpression was successful as can be seen with higher expression of PKCα in the samples where it was overexpressed (**Figures S7A, B**). The PKCα overexpression rescued the loss of IL-17A obtained by MIAT silencing (**Figure 4H**), confirming that indeed the effect of MIAT on IL17A expression was at least in part mediated through downregulation of PKCα expression.

### STAT3 and MIAT Have Overlapping and Unique Targets During Th17 Differentiation

**Figure 5:**
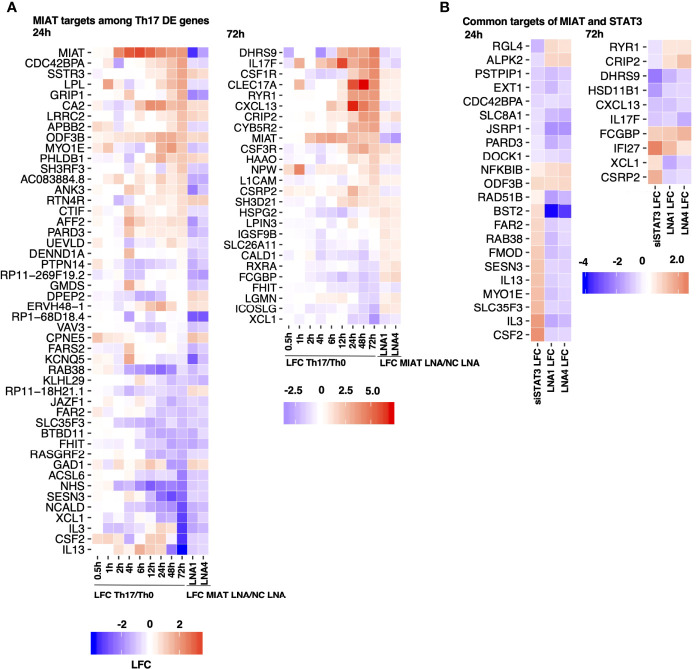
STAT3 and MIAT Have Overlapping and Unique Targets during Th17 Differentiation. **(A)** A subset of MIAT-regulated genes that are DE also during human Th17 cell differentiation (7). 24- (left) and 72-h (right) MIAT-regulated genes are plotted separately. The first nine columns show the log fold-change (LFC) of expression between Th17 and Th0 at indicated time points. The remaining two columns show the LFC between MIAT LNA1/4- and NC-treated Th17 samples. **(B)** Heatmaps represent MIAT target genes that were also regulated by STAT3 siRNA treatment from our previous study (15). Both 24 (left) and 72 h (right) MIAT targets are plotted. The first column shows the LFC of genes upon STAT3 silencing, and the remaining columns show LFC of genes upon MIAT silencing.

To investigate the expression of Th17-related genes in MIAT silenced cells, we overlaid MIAT-regulated genes with genes that were DE during the 72 h of human *in-vitro* Th17 differentiation in our previous study (7). During Th17 differentiation, 48 and 26 MIAT targets were DE at 24 h and 48 h, respectively (**Figure 5A**). Many Th17-upregulated genes, e.g., IL17F, CSF2, CXCL13, and PRKCA, were downregulated upon MIAT silencing. On the other hand, the genes downregulated in Th17 cells, e.g., RXRA and ICOSLG, were upregulated upon MIAT deficiency (**Figure 5A**).

We overlaid MIAT targets identified in this study with STAT3 targets in Th17 cells from our earlier report (15). Both MIAT and STAT3 regulate 32 genes at both time points (**Figure 5B**). Most genes downregulated by STAT3 silencing (e.g., CXCL13 and IL17F) were also downregulated by MIAT silencing. However, most genes upregulated by STAT3 silencing (e.g., IL3 and IL13) were downregulated by MIAT silencing (**Figure 5B**).

Among the similarly regulated genes, four (i.e., NFKBIB, ODF3B, FCGBP, and IFI27) were upregulated, whereas 11 (i.e., PSTPIP1, EXT1, CDC42BPA, SLC8A1, JSRP1, PARD3, DOCK1, DHRS9, HSD11B1, CXCL13, and IL17F) were downregulated by STAT3 and MIAT silencing. The upregulation of NFKBIB in MIAT- and STAT3-silenced cells is consistent with decreased Th17 differentiation as the gene product inhibits NF-κB signaling, which is required for Th17 development (30).

Among the genes regulated in the opposite direction, four (i.e., RGL4, ALPK2, RYR1, and CRIP2) were downregulated by STAT3 silencing and upregulated by MIAT silencing, whereas 13 (i.e., RAD51B, BST2, FAR2, RAB38, FMOD, SESN3, IL13, MYO1E, SLC35F3, IL3, CSF2, XCL1 and CSRP2) were upregulated by STAT3 silencing and downregulated by MIAT silencing. In a recent study, IL-3 was identified as a marker of encephalitogenic T cells but was not required for pathogenicity (31). IL-13 is a negative regulator of IL-17A expression (32). GM-CSF, encoded by *CSF2*, regulates pathogenic Th17 cell-mediated inflammatory autoimmunity (33).

Ingenuity Pathway Analysis (IPA) of the MIAT-regulated genes identified pathways associated with IL-17 to be enriched, further confirming that Th17-related genes are enriched among the MIAT targets (**Figure S8A**). In addition, several metabolic pathways previously associated with Th17 cell differentiation were enriched among the MIAT target genes in the IPA analysis (**Figure S8A**). The results confirm that MIAT contributes to Th17 differentiation by regulating the expression of several genes and pathways essential for Th17 differentiation.

### Th17-Specific TFs Are Enriched on the Promoters of MIAT Target Genes

To determine the regulation of target genes by MIAT and whether MIAT cooperates with certain transcription factors (TFs) during Th17 differentiation, we performed TFBS enrichment analysis at the target gene promoters, separately for 24 h and 72 h up and downregulated genes. Further, for the study of TFBS, we created a custom profile of TFBS of only those TFs expressed in T cells (14). TFBS for five TFs (i.e., IKAROS, EGR1, TFII, IRF4, and BCL3) were enriched in both up- and downregulated gene promoters at both times (**Figure S8B**). Of these five TFs, IRF4 and BCL3 were also upregulated during Th17 differentiation in humans and mice (7). EGR3 and Myc were enriched at both up- and downregulated promoters at 24 h, but at 72 h they were enriched only at downregulated gene promoters. Both TFs were downregulated during Th17 differentiation in humans and mice (7).

Interestingly, the TFBS for BCL11A, the TF specific to B cells, was enriched at both time points but only on the upregulated gene promoters. Further, BCL11A was upregulated in human but not mouse Th17 cells (7). Conversely, EGR2 was enriched on the downregulated gene promoter at both times, and it was upregulated during human Th17 cell differentiation (**Figure S8B**). Other interesting factors enriched at an early time point include HELIOS, RORγt, AP1, HIF1α, and SMAD5, and at later a time point include HIC1 and NF-κB p-65 (**Table S3**). Each of these factors contribute to T helper cell differentiation to one or more subsets. The analysis points towards a combinatorial and pleiotropic effects of MIAT during Th17 differentiation.

### MIAT Is Associated With the Autoimmune Phenotype

Given the positive regulation of proinflammatory genes (e.g., IL17, CXCL13, and CSF2) by MIAT, we were interested in investigating the significance of MIAT in autoimmunity. We used the GeneNetwork (genenetwork.nl) database that exploits data from 31,499 public RNA-seq samples to identify pathways and human phenotype ontology (HPO) for MIAT (34). Based on gene co-expression, autoantibody positivity and autoimmune disease-related phenotype were among the most statistically significant HPO terms associated with MIAT (**Figure S9A**). Besides, among the KEGG pathways, primary immunodeficiency**-**related pathway was the most significantly associated with MIAT (**Figure S9B**), suggesting that MIAT is coexpressed with genes associated with immune-related diseases.

The MIAT locus has a genetic association with RA. The locus harbors 56 SNPs that have an eQTL effect on MIAT (dice-database.org). Among those, rs1981493 is a risk SNP for RA with an odds ratio of 1.05 and a nominal P-value of 0.017 in the European population (35). The data from 377 RA patients with this SNP have been shown to have elevated MIAT expression in whole blood (36). This SNP is located in the first intron of MIAT and it resides in a TFBS for AP-1, Egr-1, NRSF, and Sin3Ak-20 (37).

### MIAT Is Highly Expressed in T Cells From Synovia of RA Patients

**Figure 6:**
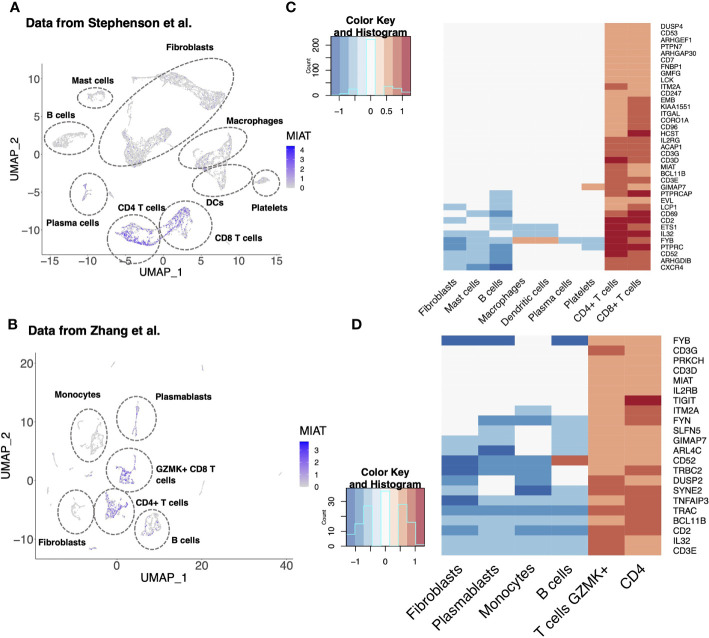
MIAT Is Highly Expressed in the Synovial T Cells of Rheumatoid Arthritis Patients. **(A, B)** MIAT expression in RA synovium. Reanalysis of scRNA-seq data of RA synovium from Stephenson et al. **(A)** and Zhang et al. **(B)** showing MIAT expression in different cell-type clusters. The expression was visualized using Uniform Manifold Approximation and Projection (*UMAP*), where each point represents a single cell. **(C, D)** Top genes co-expressed with MIAT in RA synovium. Reanalysis of scRNA-seq analysis from Stephenson et al. **(C)** and Zhang et al. **(D)**. Gene expression is displayed in log2FC. The color key indicates upregulation (red) or downregulation (blue).

RA is an autoimmune disorder of the joints in which proinflammatory cytokines, such as TNFα, IL-17A, immune cells and fibroblast-like synoviocytes, attack joints and cause bone deformation. In a scRNA-seq analysis of synovial tissue of five RA patients, MIAT was upregulated in CD4^+^ T cells (**Figure 6A**) (26). Upregulation of MIAT in T cells infiltrating RA synovia was confirmed in another scRNA-seq dataset of 21 RA patients (**Figure 6B**) (27). In the latter study, MIAT was highly expressed in T cells, followed by B cells, whereas in monocytes and fibroblasts, the expression was barely detected. Among T cells, MIAT was expressed both in CD4^+^ and CD8^+^ populations with the highest expression in CD4^+^ FOXP3^+^ Treg cells, followed by CD4^+^ PD-1^+^ T peripheral or follicular helper (Tph/Tfh) cells (**Figure S9C**). Tph cells are newly identified PD1hi CXCR5^−^ CD4^+^ cells, which are important in RA disease development (38). Among T cells, MIAT expression was lowest in CD4^+^CCR7^+^ naive and central memory T cells, suggesting MIAT is expressed upon T-cell activation. Interestingly, among the B cells, MIAT had the highest expression in plasmablasts, suggesting that MIAT is induced in activated B cells, albeit at a lower level than T cells. In general, the expression was lower in CD8 T cells than in CD4 cells.

Next, we investigated the genes co-expressed with MIAT in the two above mentioned studies (26, 27). As expected, and based on the T-cell-specific expression of MIAT, an exceptionally high fraction of the coregulated genes were T-cell-related (**Figures 6C, D**). Ten genes (i.e., BCL11B, CD2, CD3D/E/G, CD52, FYB, GIMAP7, IL-32, and ITM2A) were coregulated with MIAT in both datasets, and all 10 are involved in T-cell activation, differentiation, and effector functions.

To determine if MIAT induction is involved in other autoimmune diseases, we analyzed its expression in a microarray dataset from psoriasis patients (39). MIAT expression was higher in psoriatic lesion skin than in healthy skin. Interestingly, the treatment with brodalumab, a competitive inhibitor of the IL-17 receptor A subunit, reduced MIAT expression in psoriatic skin lesions (**Figure S10**), suggesting MIAT is involved in other Th17 related autoimmune disorders.

## Discussion

We found that MIAT is highly induced very early-on during human Th17 cell differentiation with concomitant increased chromatin accessibility at the locus. STAT3 mediates the induction of MIAT during Th17 differentiation by binding to its promoter. MIAT resides in the nucleus and regulates the expression of several key Th17 genes, including IL17A, IL17F, CCR6, and CXCL13. We propose that MIAT regulates IL17A expression by modifying the chromatin accessibility of the IL17A locus and by regulating the expression of protein kinase C alpha (PKCα), an upstream regulator of IL17A. Interestingly, a reanalysis of published data showed that MIAT was expressed in T cells from the synovium of RA patients and the skin lesions of psoriasis patients.

Recently, in a mouse model, MIAT was shown to promote Th17 response in allergic inflammation by inhibiting miR-10b-5p (11). Further, MIAT was upregulated in rats with atherosclerosis, and it aggravated the atherosclerotic damage through PI3K/Akt activation (40). On the other hand, the expression of MIAT was reduced in the colonic tissues of colitis mice, where MIAT expression was under the control of the signaling through Sphingosine 1-phosphate receptor 2 (S1P2) (12). Further work is needed to understand the function of MIAT in Th17-mediated autoimmune disease.

LincRNAs are emerging as regulators of CD4^+^ T-cell differentiation to distinct subsets (41). Interestingly, lincRNA NEAT1 is upregulated in Th17 cells and PBMC from RA patients, and NEAT1 functions as an upstream regulator of STAT3 (42). Here, we identified MIAT as a molecule regulated by STAT3 in Th17 cells. Importantly, regulation of Th17 differentiation by MIAT is probably specific to humans because the mouse homolog of the gene was not expressed during mouse Th17 cell differentiation (7). During early stages of Th17 differentiation MIAT induction appears to be primarily driven by signaling through IL-6R/STAT3. MIAT expression was much higher in cells induced to differentiate to Th17 direction compared to cells activated in the absence of Th17 polarising stimuli (**Figure 1A**). Further, increased expression of MIAT was regulated by STAT3. Yet its expression was induced to some extent after activation of cells through T cell receptor and CD28 suggesting that besides IL6/STAT3 also signals through T cell receptor/CD28 contribute to the induction of MIAT.

Based on the data presented here, the effect of MIAT on Th17 cell differentiation is in part mediated by PKCα. In the RNA-seq analysis, PKCα was downregulated early after induction of Th17 differentiation in MIAT silenced cells, and the downregulation was confirmed by western blotting and TaqMan qPCR analysis. Further, silencing PKCα expression led to reduced IL17 expression by differentiating Th17 cells, which was rescued by overexpressing PKCα, suggesting that regulation of IL17A expression by MIAT during early human Th17 differentiation is, at least partly, through PKCα. PKCα is an early gene induced upon T cell activation, and is known to positively regulate IL-17 expression in mice (29).

Among the MIAT targets that were also DE during Th17 differentiation in Tuomela et al. study (7), were two transcription regulators: glutamate receptor-interacting protein 1 (GRIP1) and retinoid X receptor-alpha (RXRA). GRIP1 was upregulated in Th17 cells and downregulated by MIAT silencing at 48 h. Conversely, RXRA was downregulated in Th17 cells and upregulated upon MIAT silencing.

TFBS for BCL11A was enriched on the promoters of genes upregulated by MIAT silencing as well as on the genomic regions differentially accessioble upon MIAT silencing. Among lymphocytes, BCL11B, the paralogue of the BCL11A, is exclusively expressed in T cells and its expression in T cell is tightly associated to T-lymphoid lineage commitment. However, BCL11A regulates development of B cells (43). Futher work is needed to study the role of BCL11A in Th17 cell differentiation.

Analysis of MIAT and STAT3 targets suggests that STAT3 and MIAT have overlapping as well as unique targets. STAT3 and MIAT both positively regulated CXCL13, whereas STAT3 and MIAT silencing oppositely regulated CSF2. Interestingly, CXCL13 and CSF2 have been associated with autoimmune diseases (33, 44). CXCL13 levels in the synovial fluid of patients with RA correlate with IL-17 levels (45). Further, human alloreactive and pathogen-specific Th17, but not Th1 or Th2, clones express CXCL13 (45). CSF2 is essential for pathogenic Th17 cells and inflammatory autoimmune diseases (33). Interestingly, MIAT has particularly high expression in brain tissues, whereas STAT3 is constitutively expressed in many tissues except the brain (https://gtexportal.org/). Overlapping and dissimilar targets of MIAT and STAT3, along with their unique expression profiles, may provide an opportunity for modulation of Th17 cell response by targeting STAT3 and MIAT.

MIAT induction is apparently specific for human Th17 cells during the early stages of differentiation. However, in fully differentiated human cells *in-vivo*, MIAT is quite highly expressed in other T cell subsets including regulatory T cells and follicular helper T cells (https://dice-database.org). Indeed, in the reanalysis of the scRNA-seq data from synovial tissues of RA patients, MIAT expression was specific for T cells, but not restricted to Th17 cells.

RA is characterized by accumulation of inflammatory cells in the synovia (46). The cytokine secreted by Th17 cells, IL-17A, along with TNFα are the critical inflammatory cytokines implicated in the pathogenesis of RA, and the current treatment includes their antagonists (47). However, blocking TNFα receptor or the cytokine carries an increased risk of infections (48) and lymphoma (49). Further, strategies for neutralizing proinflammatory cytokine IL17A are less effective in RA (50). Understanding the contribution of MIAT in the regulation of Th17 response may offer new approaches to mitigate the inflammation in RA given its connection with IL-17 and its co-regulation with IL-32, another cytokine increasingly associated with autoimmune diseases, including RA.

## Data Availability Statement

Raw and processed RNA-seq and ATAC-seq data can be accessed via GEO database at NCBI with accession numbers GSE183664 and GSE183918, respectively.

## Ethics Statement

The study was reviewed and approved by Hospital District of Southwest Finland. Ethics Committee of Hospital District of Southwest Finland approved usage of the umbilical cord blood samples from unknown donors.

## Author Contributions

MMK and MHK designed and performed the experiments, analyzed data, prepared figures, and wrote the manuscript; UUK designed experiments, analyzed data, prepared figures, and wrote the manuscript; SK, NP and SJ reanalyzed single-cell RNA-seq data and prepared figures. LK analyzed RNA-seq data and helped in experimental planning. OR designed experiments and wrote the manuscript. LE supervised SK and SJ and provided scientific expertise. RL designed and supervised the study and wrote the manuscript. All the authors commented and approved the manuscript.

## Funding

MMK was supported by the University of Turku graduate school on Turku Doctoral Programme of Molecular Medicine and a central grant from the Finnish Cultural Foundation. RL was supported by the Academy of Finland, AoF, Centre of Excellence in Molecular Systems Immunology and Physiology Research (2012-2017) grant 250114; by the AoF grants 292335, 294337, 292482, 319280, 329277, 331793, 335435 and 31444; by grants from the JDRF; the Novo Nordisk Foundation (grant NNF19OC0057218); the Sigrid Jusélius Foundation; Jane and Aatos Erkko Foundation and the Finnish Cancer Foundation. LLE reports grants from the European Research Council ERC (677943), European Union’s Horizon 2020 research and innovation programme (675395), Academy of Finland (296801, 304995, 310561, 314443, and 329278), Juvenile Diabetes Research Foundation JDRF (2-2013-32), and Sigrid Juselius Foundation, during the conduct of the study. Our research is also supported by University of Turku, Åbo Akademi University, Turku Graduate School, InFLAMES Flagship Programme of the Academy of Finland (decision number: 337530), Biocenter Finland, and ELIXIR Finland.

## Conflict of Interest

The authors declare that the research was conducted in the absence of any commercial or financial relationships that could be construed as a potential conflict of interest.

## Publisher’s Note

All claims expressed in this article are solely those of the authors and do not necessarily represent those of their affiliated organizations, or those of the publisher, the editors and the reviewers. Any product that may be evaluated in this article, or claim that may be made by its manufacturer, is not guaranteed or endorsed by the publisher.
