# Supplementary material for: Long Intergenic Noncoding RNA MIAT as a Regulator of Human Th17 Cell Differentiation
Source: Front Immunol. 2022 Jun 15;13:856762. doi: 10.3389/fimmu.2022.856762 (PMC9242727; doi:10.3389/fimmu.2022.856762)
Supplement: Supplementary file 1 [file DataSheet_1.docx]

**Supplementary Methods**

The following LNAs (provided by EXIQON/QIAGEN) were used to silence MIAT:

LNA-NC (non-targeting control): 5´-AACACGTCTATACGC -3´;

LNA-1: 5'-ACTTTGGCATTCTAGG-3´;

LNA-2: 5´-AAGTGCTGAAACTGAG-3´

LNA-4: 5'-AGGCTGGTAGGCTATG-3´.

The following siGENOME SMARTpool siRNA (provided by Dharmacon) were used for PRKCA silencing:

5´-GGAUUGUUCUUUCUUCAUA-3´;

5´-GAAGGGUUCUCGUAUGUCA-3´;

5´-GGACUGGGAUCGAACAACA-3´;

5´-UAAGGAACCACAAGCAGUA-3´;

The NT siRNAs (synthesized by Sigma) were used:

siNT 5´-AAUUCUCCGAACGUGUCACGU-3´

**TaqMan qPCR**

The following primers used in this study:

*IL17A*

Universal Roches Probe library probe #8
Forward primer 5`-TGGGAAGACCTCATTGGTGT-3´;
Reverse primer 5`-GGATTTCGTGGGATTGTGAT-3´;
*MIAT*
Universal Roches Probe library probe #75

Forward primer 5`-GAGTGTGTGTGCATCTTGACAAT-3´;
Reverse primer 5`-GAGGGGTCGAAGAGAATGTG-3´
*STAT3*
Probe (FAM-TAMRA): 5'-TCGGCCAGAGAGCCAGGAGCAT-3'

Fw: 5'-GAGGAGGCATTCGGGAAGTATT-3'

Rev: 5'-GCGCTACCTGGGTCAGCTT-3'
*PRKCA*

Universal Roches Probe library probe #202
Forward primer 5’-TGGCGTCCTGTTGTATGAAA-3´;
Reverse primer 5’-CGTTGTGCTCCATGATAGACTG-3´;

*RORC*

Universal Roche Probe library probe #87

Forward primer: 5’- AGACTCATCGCCAAAGCATC -3’

Reverse primer: 5’- TCCACATGCTGGCTACACA -3’

*EEF1A1* (EF1α)

Probe (FAM-TAMRA): 5' -AGCGCCGGCTATGCCCCTG- 3'

Forward primer: 5' -CTGAACCATCCAGGCCAAAT- 3'

Reverse primer: 5' -GCCGTGTGGCAATCCAAT- 3'

**ChIP-PCR primers**

STAT3 ChIP primers on MIAT promoter

Forward primer sequence (5'->3'): AAGTGTCCGGAAATGGTTTG

Reverse primer sequence (5'->3'): CGCCCCTCAACTTTCTAGG

**ATAC-PCR**

DNA sequences of the regions and the primers used to TaqMan ATAC-qPCR are as follows:

**Region 1:** hg38_dna range=chr6:52186481-52186673

Forward primer sequence (5'->3'): GCATGAAACGCTGGGTTCTG

Reverse primer sequence (5'->3'): TCTTGAAAATGCTGCACAATGACT

**Region 2:** hg38_dna range=chr6:52186432-52186721

Forward primer sequence (5'->3'): TCCTGCACTAACGTGCGATG

Reverse primer sequence (5'->3'): CTCCATAGTCAGAACCCAGCG

**Region 3:** hg38_dna range=chr6:52186635-52186787

Forward primer sequence (5'->3'): CAAGAACTGATACACAGCAGTTTGA

Reverse primer sequence (5'->3'): CTTCTTGTGTGGTTTAGCCCC

**Human CD4+ T cell isolation, activation, and differentiation**

CD4+ T cells were isolated from umbilical cord blood as described previously earlier (1–4). Human umbilical cord blood was layered on ficol (GE Healthcare, cat# 17-1440-03) for isolation of white blood cells. CD4+ T cells were then isolated using the bead-based CD4+ isolation kit from Invitrogen (cat# 11331D). For activation of T cells, a combination of plate-bound anti-CD3 (3750 ng/6-well culture plate well) (Beckman Coulter, cat# IM-1304) and soluble anti-CD28 (1 mg/mL)(Beckman Coulter, cat# IM1376) antibodies were used.

For Th17 cell differentiation, isolated CD4+ cells were activated with a combination of plate-bound anti-CD3 (750 ng/24-well culture plate well; Immunotech/Beckman Coulter REF # IM-1304) and soluble anti-CD28 ((1ug/mL; Immunotech/Beckman coulter REF # IM1376) antibodies in serum-free X-Vivo 20 medium (Lonza), in the absence (Th0) or presence (Th17) of IL-6 (20ng/ml, Roche, Cat# 11138600 001); IL-1b (10ng/ml, R&D Systems Cat # 201 LB); TGF-b1 (10ng/ml, R&D Systems Cat# 240); anti-IL-4 (1 mg/ml) R&D Systems Cat# MAB204) and anti-IFN-g (1 mg/ml R&D Systems Cat#MAB-285). Differentiation of Th17 cells was confirmed by measuring IL-17 expression by quantitative real-time PCR, at 72 hours of Th17/Th0 culturing (2).

For iTreg cell culturing, after CD25+ cells were depleted using LD columns from CD25 depletion kit (Miltenyi Biotec), CD4+CD25 cells were activated with plate-bound anti-CD3 (500 ng/24-well culture plate well) and soluble anti-CD28 (500 ng/mL) at a density of 2 3 106 cells/mL of X-vivo 15 serum-free medium (Lonza). For iTreg differentiation, the medium was supplemented with IL-2 (12 ng/mL), TGF-b (10 ng/mL) (both from R&D Systems), all-trans retinoic acid (ATRA) (10 nM; Sigma-Aldrich), and human serum (10%) and cultured at 37C in 5% CO2. Control Th0 cells were stimulated with plate-bound anti-CD3 soluble anti-CD28 antibodies without cytokines. For confirmation of iTreg cell differentiation, we used intracellular staining to measure, at 72 hours of iTreg culturing, expression of FOXP3 which is the major transcription factor driving Treg differentiation. Intracellular staining was performed using buffer sets of Human Regulatory T cell Staining Kit (eBioscience/Thermo Fisher Scientific), following the manufacturer’s pro- tocol. The following antibodies were used: anti-human FOXP3-PE (eBioscience, Cat. No. 12-4776-42) and rat IgG2a isotype control (eBioscience, Cat. No. 72-4321-77A). All samples were acquired by a flow cytometer (LSRII) and analyzed either with FlowJo (FLOWJO, LLC) or with Flowing Software (4).

Th1 and Th2 cell differentiation were done as described previously (5). Briefly, purified naive CD4+ T cells were activated with plate-bound anti-CD3 (500 ng/24-well culture plate well) and 500 ng/ml soluble anti-CD28 and cultured in the absence (Th0) or presence of 2.5 ng/ml IL-12 (R&D Systems) (Th1) or 10 ng/ml IL-4 (R&D Systems) (for Th2). At 48 hours following the activation of the cells, 17 ng/ml IL-2 (R&D Systems) was added to the cultures. Differentiation of Th1 and Th2 cells was confirmed by measuring (using flow cytometry) the expression of T-bet and Gata3 at 72 hours after cell activation. Briefly, cells were fixed and permeabilized using the Intracellular Fixation & Permeabilization Buffer Set (eBioscience/Thermo Fisher Scientific), according the manufacturer’s protocol. The following antibodies were used: anti-human GATA3-PE (eBioscience, 12-9966), anti-human T-bet-BV711 (BD, 563320) and corresponding isotype controls (BV711 Mouse IgG1, BD, 563044 and PE Rat IgG2b, eBioscience, 12-4031-82). Samples were acquired by BD LSRFortessa cell analyzer and data were analyzed using FlowJo software (FLOWJO, LLC).

**RNA-seq analysis**

RNA was isolated with (RNeasy Mini Kit, cat# 74106, QIAGEN) and treated in-column with DNase (RNase-Free DNase Set; QIAGEN) for 15 min. The removal of genomic DNA was ensured by an additional treatment of the samples with DNase I (Invitrogen). After RNA quantification (using Nanodrop 2000) and quality control (using BioRad Experion or Agilent Bioanalyzer), libraries for RNA-Seq were prepared using Illumina TruSeq® Stranded mRNA Sample Preparation Guide (part #15031047). The quality of the libraries was confirmed with Advanced Analytical Fragment Analyzer, and the concentrations of the libraries were quantified with Qubit® Fluorometric Quantitation (Life Technologies). Sequencing was performed at the Finnish Functional Genomics Centre using the HiSeq2500 Next-Generation Sequencing platform. FastQC was used to check the quality of the raw sequencing reads (6), and Trimmomatic (7) was used to trim the adapters and low-quality bases. Tophat2 (8) was used to align the trimmed reads to the human reference genome GRCh37.75 (Ensembl release 75). HTseq-count (9) was used to calculate the summarized read counts for each gene. The differentially expressed genes were identified using the R/Bioconductor package DEseq (10). To select top genes, we exploited the differential knockdown efficiency of the two LNAs. DE genes were ranked according to their similarity with MIAT expression upon knockdown with two different LNAs. Genes that were more efficiently downregulated or upregulated with more efficient knockdown were ranked higher.

**RNAScope *In Situ* Hybridization (ISH)**

RNAScope experiments were performed using RNAScope multiplex fluorescent reagent kit v2 ACD a biotechne brand (cat# 323100), following manufacturer’s recommendations. Briefly, cells were cultured on the sterilized glass coverslips. The medium was removed, and the cells were washed once with PBS. Next, 10% neutral buffered formalin was added in each well and incubated for 30 min at room temperature (RT), followed by two washes with PBS. The PBS was removed, and the cells were then dehydrated with 50, 70, and 100% ethanol at RT for 5, 5, and 10 min, respectively. A hydrophobic barrier (from the RNAScope kit) was drawn two to four times around each coverslip using the manufacturer’s hydrophobic barrier pen. The coverslips were then left to dry at RT. The coverslips were then washed once with PBS and incubated with protease III plus (RNAScope Kit) for 10 min at RT, followed by two washes with PBS.

The cells were incubated with the MIAT RNAScope probes (cat# 458501 ) at 40° C for 2 h. 2-4 drops of the probes were added so that they completely covered the coverslip. The cells were washed with wash buffer provided in the kit. Probes were hybridized in AMP1 (RNAScope Kit) for 30 min at 40° C, and then the cells were washed with the wash buffer. The procedure was repeated with AMP2 and AMP3, where the incubation time with AMP3 was 15 min, followed by washing with wash buffer for 2 min. For development of the HRP-C1 signal, the wash buffer was removed. The cells were incubated with RNAScope® Multiplex FL v2 HRP-C1 for 15 min at 40° C. After 2x washing the cells at RT for 2 min, the cells were incubated with diluted TSA plus fluorescein (Perkin Elmer) for 30 min at 40° C.

After washing the cells twice with wash buffer at RT for 2 min, the cells were incubated with RNAScope® Multiplex FL v2 HRP blocker for 15 min at 40°C followed by two washes with wash buffer. The cells were then mounted using a Vecta shield DAPI hard-set mounting media (cat# H-1500). RNAScope images were acquired with a 3i Marianas Spinning disk confocal microscope, fitted with Yokogawa CSU-W1 scanner on an inverted Zeiss Axio Observer Z1 microscope controlled by SlideBook 6 (Intelligent Imaging Innovations), ORCA- Flash4.0 v2 sCMOS Camera (Hamamatsu Photonics), and a Plan Apochromat 100×1.4 NA oil objective. The number of cells in each image varied between 7 to 37 and, we have quantified 482 cells in Th0 condition and 554 cells in Th17 condition of all the three replicates, in each replicate there were 10 images acquired. The imaging was performed after 24 hours of culturing in Th0 and Th17 polarizing conditions. RNAScope data is analysed to plot the number of lncRNA MIAT puncta in each image in Th0 and Th17 conditions of the three replicates. The number of cells and puncta in each image were counted manually using imageJ.

**Generating PRKCA in-vitro transcribed (IVT) RNA and its overexpression**

T7 promoter containing plasmids containing either PRKCA ORF (which was commercially synthesized by GeneScript where the PRKCA ORF sequence was cloned into NheI-KpnI cloning site of pcDNA3.1(+)), or GFP sequence (empty pGEM-GFP64A plasmid) were used as template in the IVT reaction for generating PRKCA or GFP mRNAs, respectively. Plasmids were first linearized using restriction digestion enzymes: spe1 (NEB, Cat. no. R0133) was used for GFP and EcoR1 (NEB Cat. no. R0101 ) for the PRKCA plasmid. In the following step, IVT RNA was generated using T7 mScript Standard mRNA Production System (CELLSCRIPT, cat#C-MSC100265) following the manufacturer’s instructions. In the last step, the IVT RNA was incubated overnight at -80◦ C with 2.5M Lithium chloride (LiCl). The next day, IVT RNA in LiCl was centrifuged and the pellet was washed two times with 70% ethanol. The RNA pellet was then resuspended in nuclease-free water. An Agilent Bioanalyzer or BioRad Experion was used to confirm the size of the RNA at this stage. The RNA was then Capped and polyadenylated according to the instructions from the T7 mScript kit. In the final step, LiCl precipitation was repeated as described above, and the pellet was resuspended in nuclease-free water. The final IVT-generated RNA concentration was measured using Nanodrop™ 2000 (Thermo Scientific) and stored at -80◦C.

The CD4+ cells were isolated and nucleofected with NT LNA or MIAT LNA1 and were rested for 48 h in RPMI 1640 (Sigma-Aldrich) medium supplemented with 10% FCS, 50 U/mL penicillin, 50 μg/mL streptomycin, and 2 mM L-glutamine. Next, the cells were activated in Th17 polarizing conditions for 72 h, after which they were re-nucleofected with 28 picomoles of GFP or PRKCA IVT-mRNA or mock nucleofected.. The cells were allowed to rest in RPMI 1640 (Sigma-Aldrich) medium supplemented with 10% FCS, 50 U/mL penicillin, 50 μg/mL streptomycin, 2 mM L-glutamine, and 17ng/ml IL2 (Cat# 101-IL; R&D systems) for 24 hours followed by activation in Th17 polarizing media. After six hours of re-nucleofection of IVT RNA, the overexpression was checked by FACS. The IL17 ELISA samples were collected after 72 hours.

**Reanalysis of Published Single-Cell RNA-seq Data**

We examined the expression of lincRNA MIAT in immune cells from synovial tissue samples of RA patient, utilizing published single-cell RNA-seq (scRNA-seq) data (Stephenson et al., 2018; Zhang et al., 2019). The scRNA-seq data from Stephenson et al. was accessed through dbGaP (phs001529.v1.p1), and scRNA-seq data from Zhang et al. was retrieved from ImmPort (SDY998). Both datasets were preprocessed similarly. Cells with <500 genes detected with at least one fragment or >25% of molecules coming from mitochondrial genes were discarded. Genes with only zero values were filtered out. The preprocessed expression matrices were used as input for our in-house developed clustering tool ILoReg with default settings (Smolander et al., 2020). The identified clusters were visualized using uniform manifold approximation and projection (UMAP). To annotate the clusters, the marker genes for each cell type were obtained from the original publications. For identifying genes co-expressed with MIAT in a cell type-specific manner, we utilized the FindAllGeneMarkers function in the ILoReg tool to simultaneously identify all genes showing cell type-specific expression patterns. Genes that co-expressed with MIAT, i.e. had a similar expression profile in different cell types (log2 fold change ≥ 0.5, Bonferroni adjusted p-value < 0.01), were examined in the datasets. The fold change was calculated as gene’s expression in a given cell type compared to all other cell types and the significance of the differences was determined using Wilcoxon rank-sum test.

**References**

1. Elo LL, Jarvenpaa H, Tuomela S, Raghav S, Ahlfors H, Laurila K, et al. Genome-wide profiling of interleukin-4 and STAT6 transcription factor regulation of human Th2 cell programming. Immunity. 2010 Jun;32(6):852–62.

2. Khan MM, Ullah U, Khan MH, Kong L, Moulder R, Välikangas T, et al. CIP2A Constrains Th17 Differentiation by Modulating STAT3 Signaling. iScience. 2020;23(3).

3. Tripathi SK, Chen Z, Larjo A, Kanduri K, Nousiainen K, Äijo T, et al. Genome-wide Analysis of STAT3-Mediated Transcription during Early Human Th17 Cell Differentiation. Cell Rep. 2017;19(9):1888–901.

4. Ubaid Ullah, Andrabi SBA, Tripathi SK, Dirasantha O, Kanduri K, Rautio S, et al. Transcriptional Repressor HIC1 Contributes to Suppressive Function of Human Induced Regulatory T Cells. Cell Rep. 2018;22(8).

5. Hawkins RD, Larjo A, Tripathi SK, Wagner U, Luu Y, Lönnberg T, et al. Global chromatin state analysis reveals lineage-specific enhancers during the initiation of human T helper 1 and T helper 2 cell polarization. Immunity [Internet]. 2013;38(6):1271–84. Available from: http://www.ncbi.nlm.nih.gov/entrez/query.fcgi?db=pubmed&cmd=Retrieve&dopt=AbstractPlus&list_uids=23791644%5Cnhttp://www.sciencedirect.com/science/article/pii/S1074761313002379

6. Andrews S. FastQC: A Quality Control Tool for High Throughput Sequence Data [Online]. Available online at: http://www.bioinformatics.babraham.ac.uk/projects/fastqc/ [Internet]. 2010. Available from: https://qubeshub.org/resources/fastqc

7. Bolger AM, Lohse M, Usadel B. Trimmomatic: A flexible trimmer for Illumina sequence data. Bioinformatics. 2014;30(15):2114–20.

8. Kim D, Pertea G, Trapnell C, Pimentel H, Kelley R, Salzberg SL. TopHat2: accurate alignment of transcriptomes in the presence of insertions, deletions and gene fusions. Genome Biol. 2013 Apr;14(4):R36-2013-14-4-r36.

9. Anders S, Pyl PT, Huber W. HTSeq-A Python framework to work with high-throughput sequencing data. Bioinformatics. 2015;31(2):166–9.

10. Anders S, McCarthy DJ, Chen Y, Okoniewski M, Smyth GK, Huber W, et al. Count-based differential expression analysis of RNA sequencing data using R and Bioconductor. Nat Protoc. 2013 Sep;8(9):1765–86.
