# Supplementary figures and images for: Long Intergenic Noncoding RNA MIAT as a Regulator of Human Th17 Cell Differentiation
Source: Front Immunol. 2022 Jun 15;13:856762. doi: 10.3389/fimmu.2022.856762 (PMC9242727; doi:10.3389/fimmu.2022.856762)

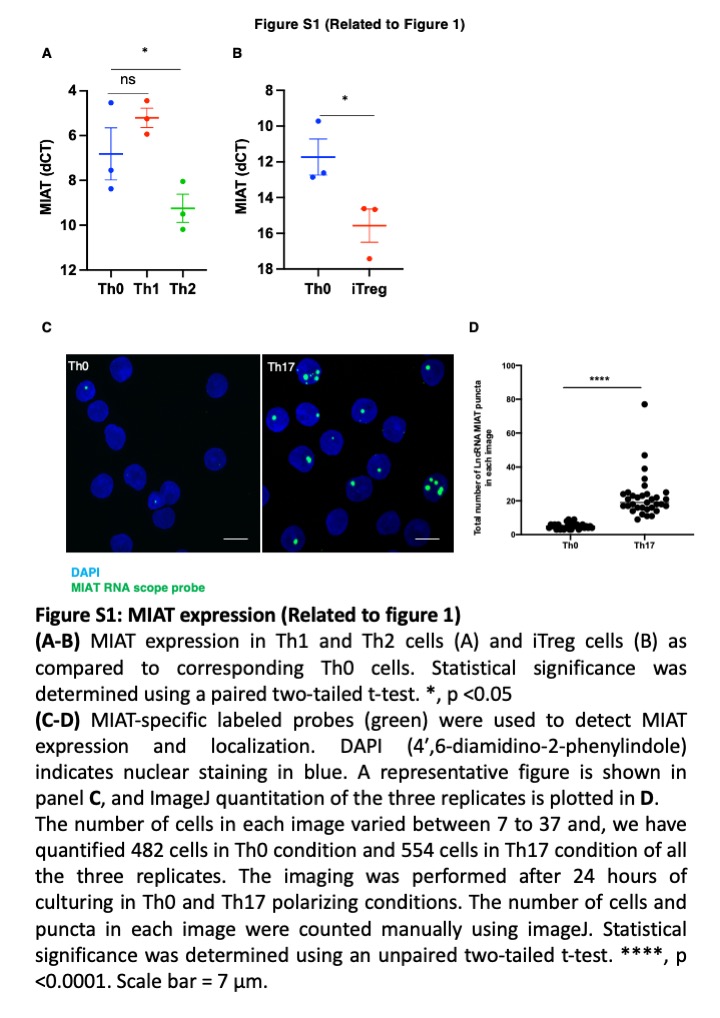

Supplement: Supplementary file 2 [file Image_1.jpg]

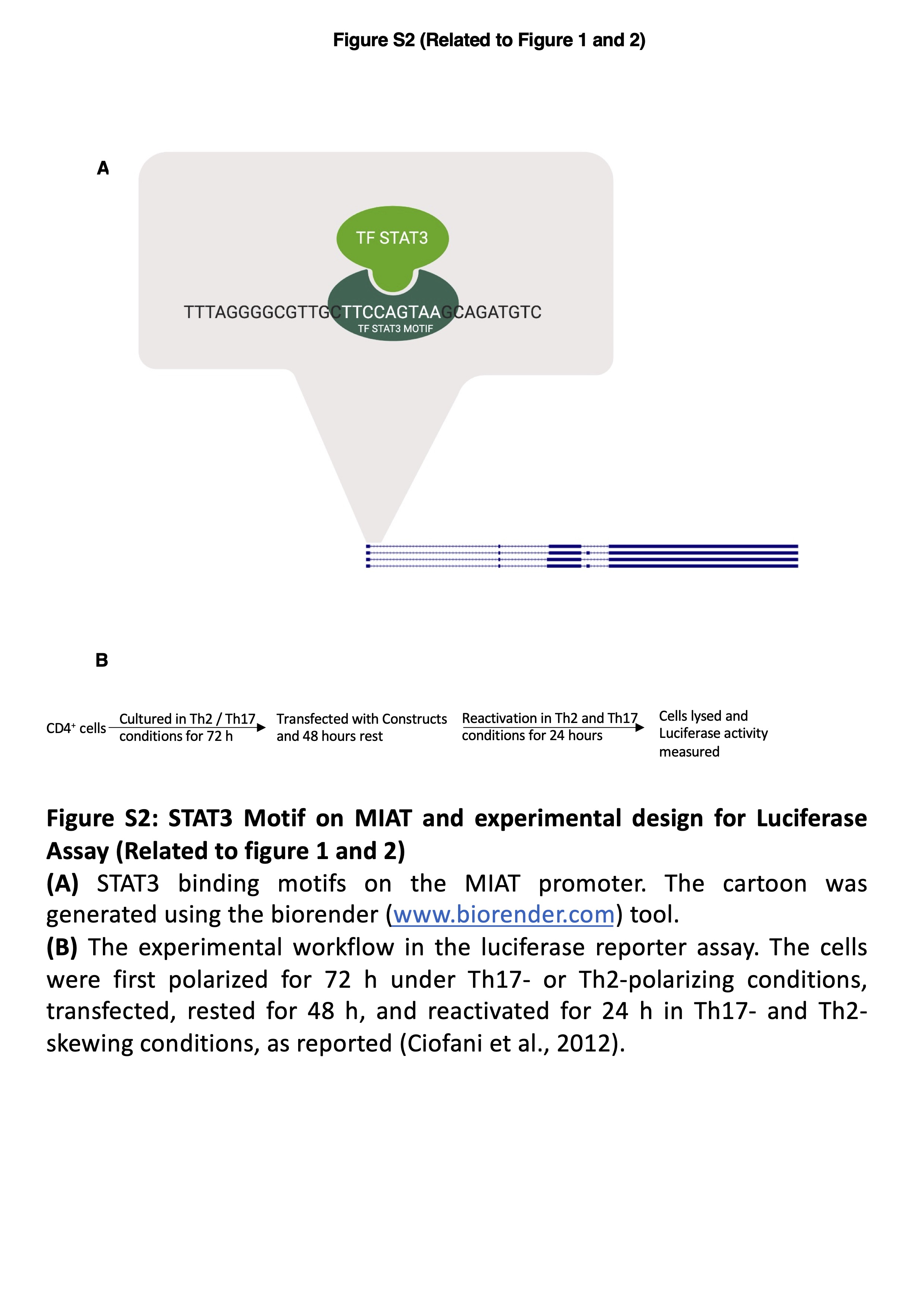

Supplement: Supplementary file 3 [file Image_2.jpeg]

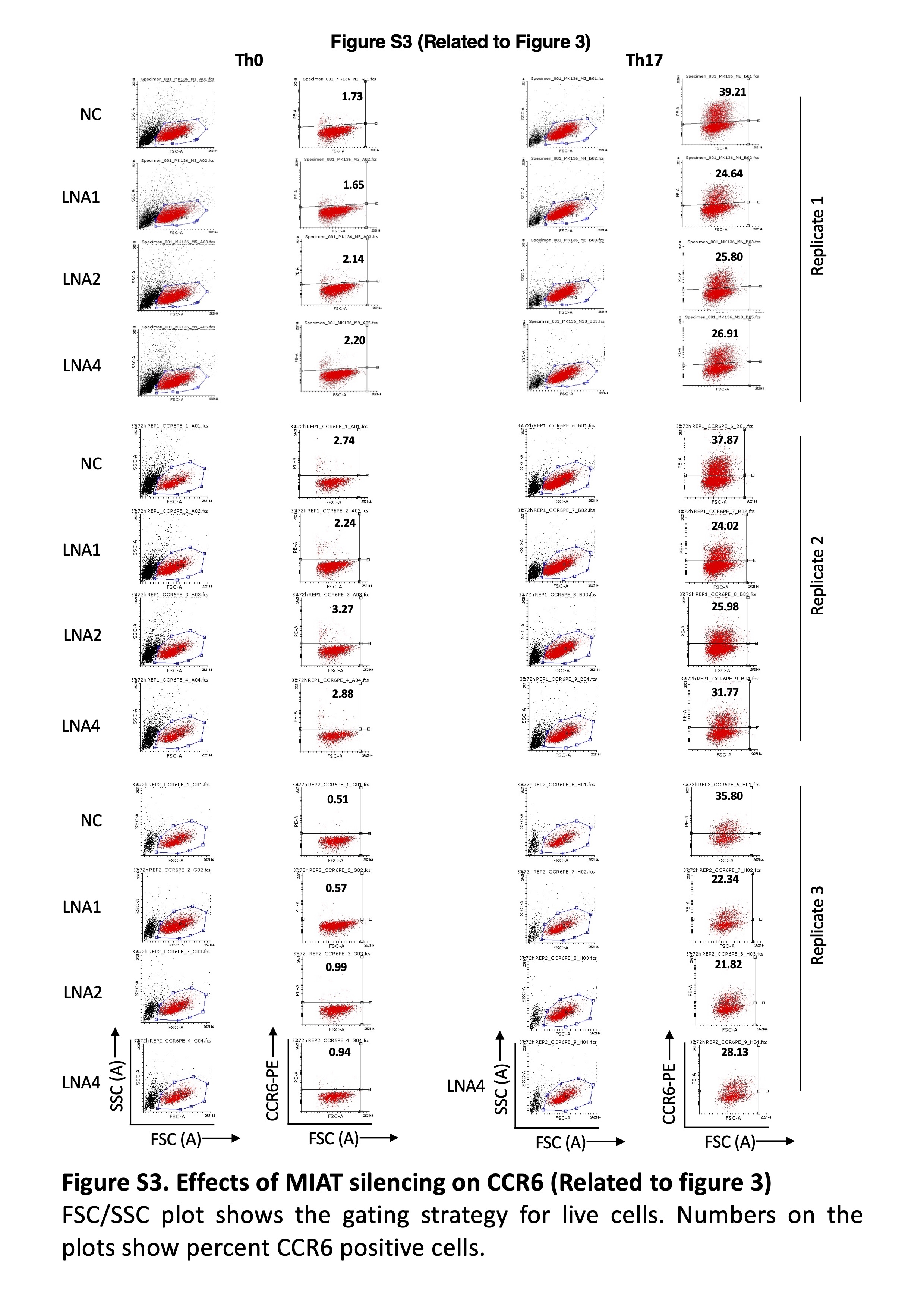

Supplement: Supplementary file 4 [file Image_3.jpeg]

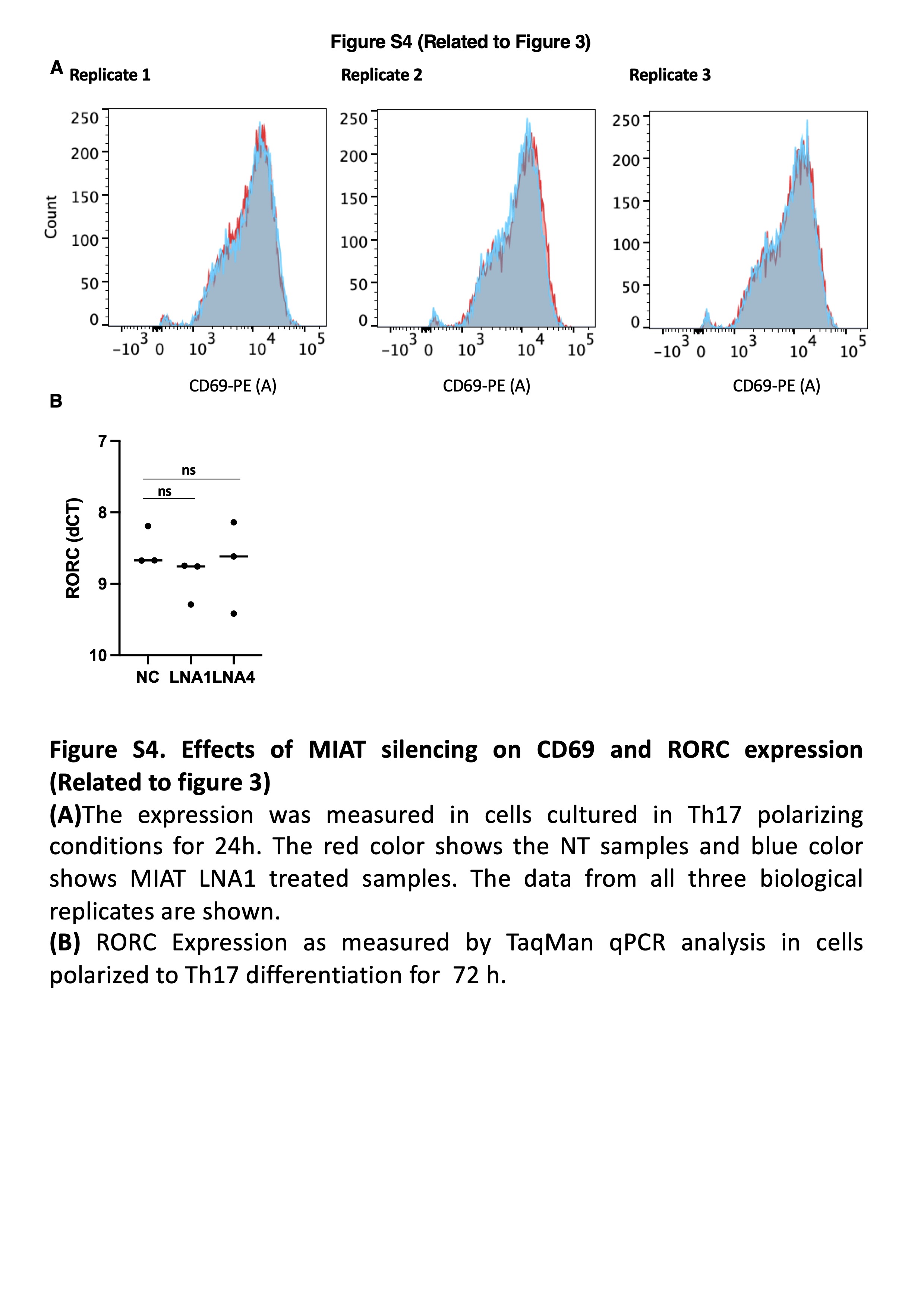

Supplement: Supplementary file 5 [file Image_4.jpeg]

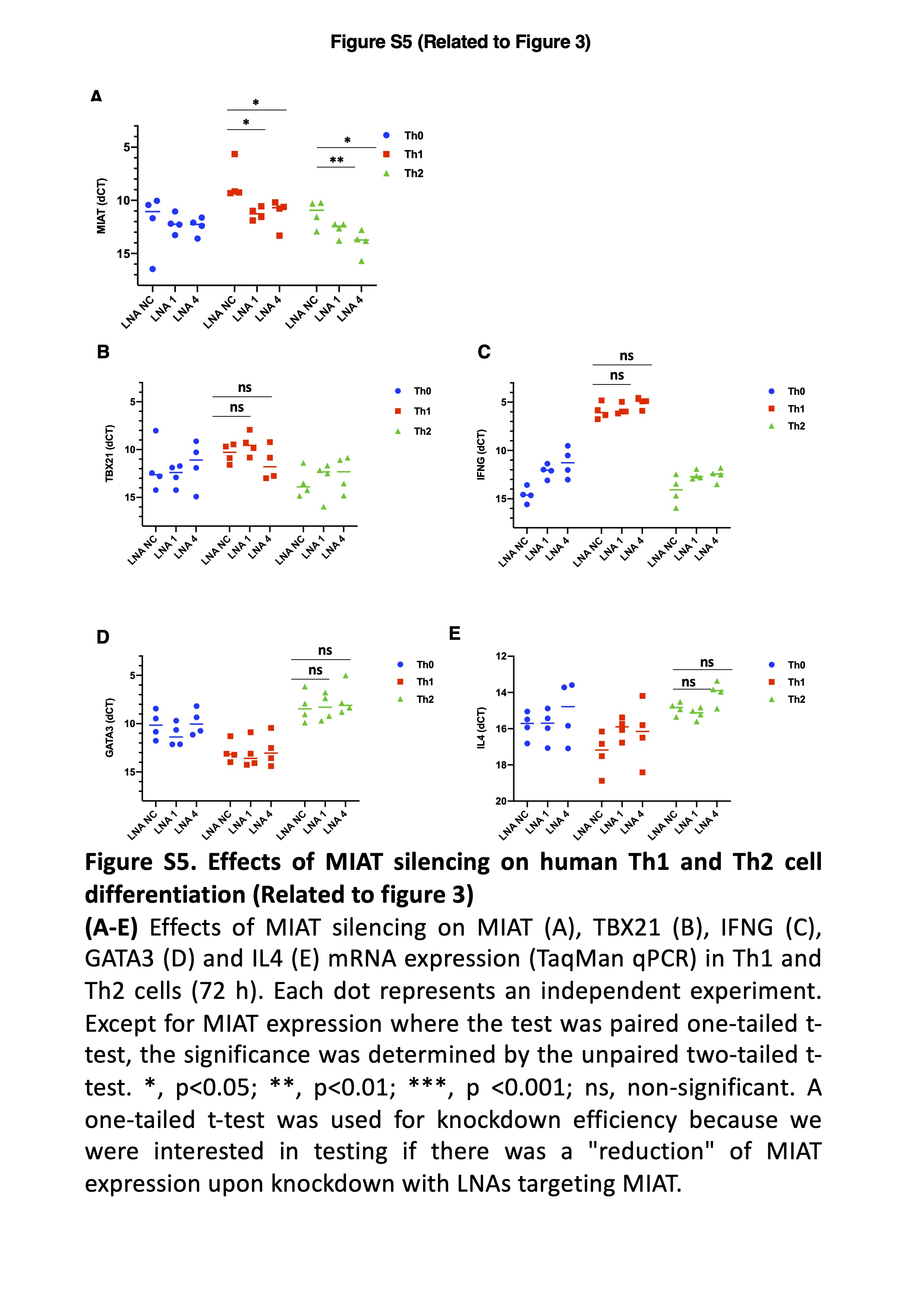

Supplement: Supplementary file 6 [file Image_5.jpeg]

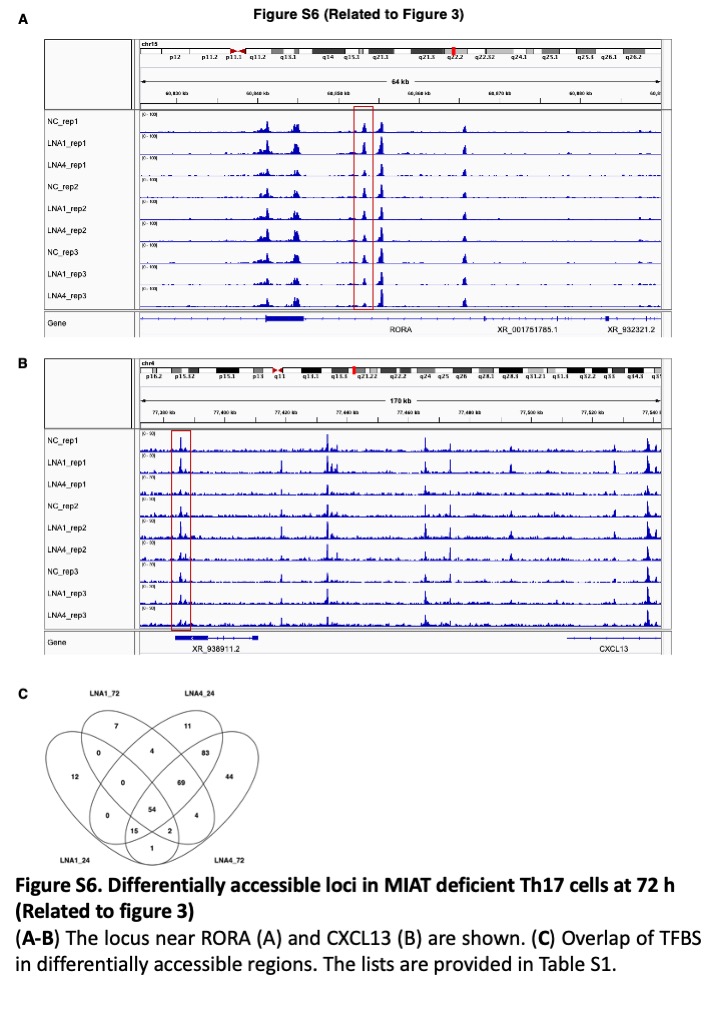

Supplement: Supplementary file 7 [file Image_6.jpg]

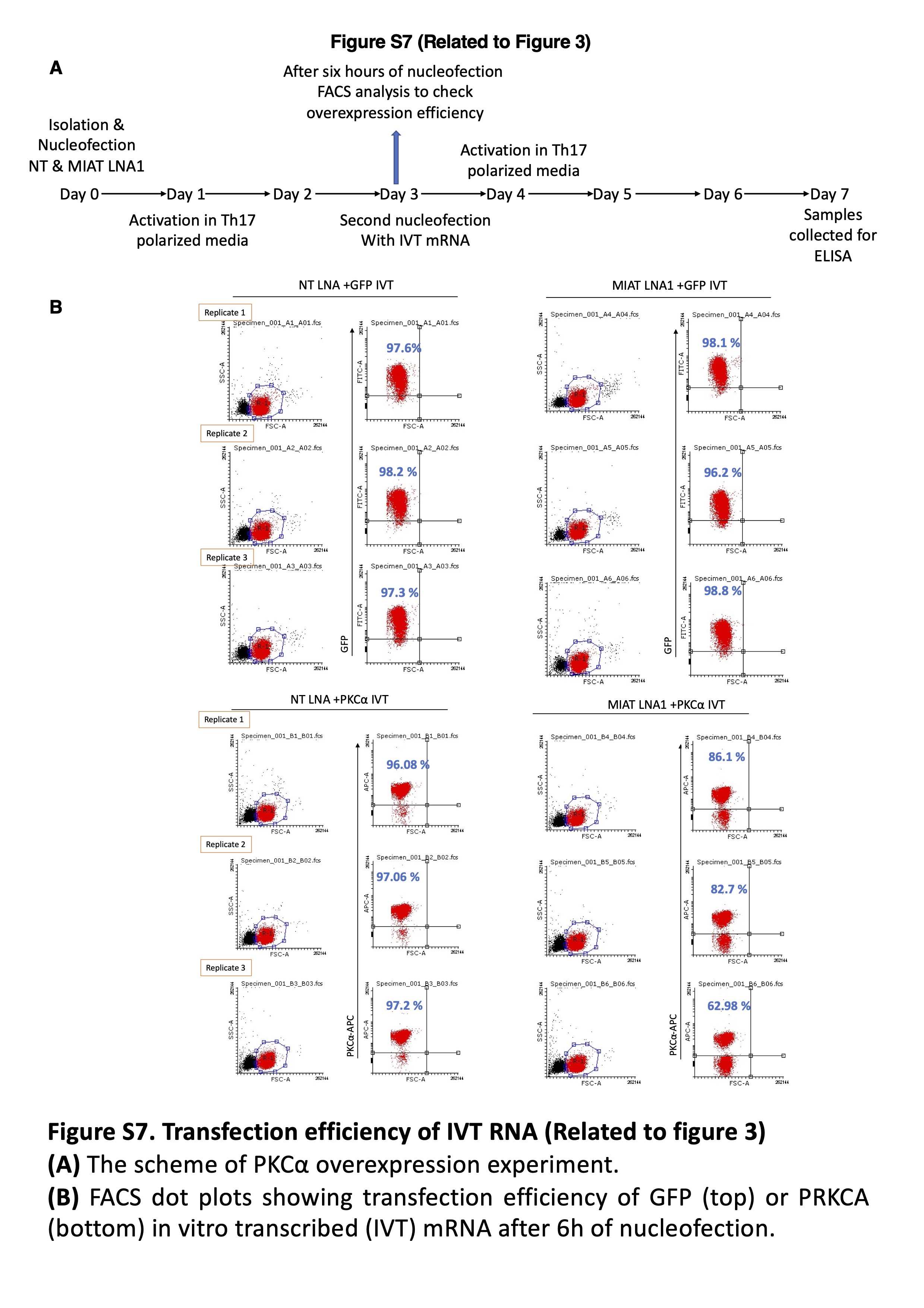

Supplement: Supplementary file 8 [file Image_7.jpeg]

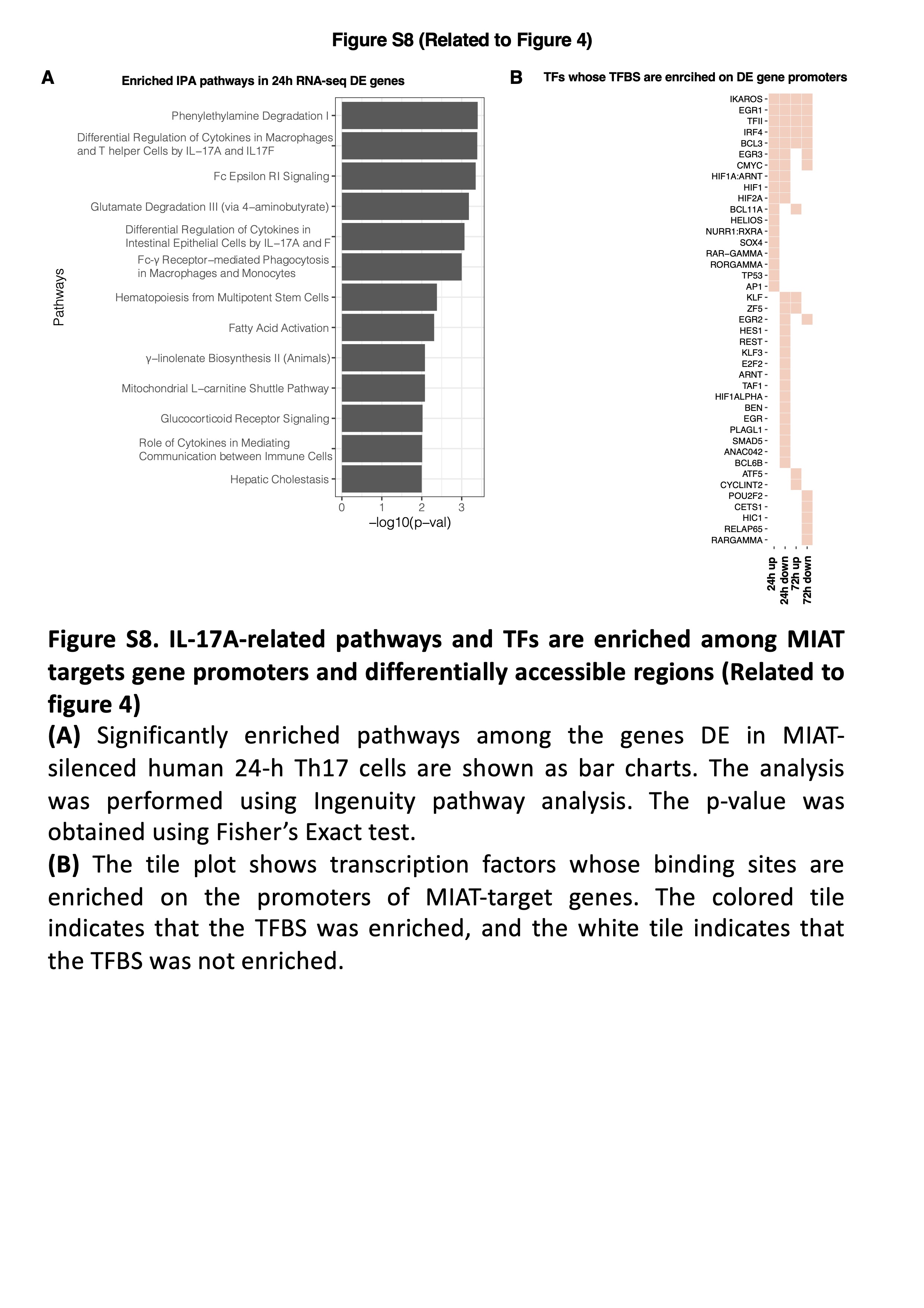

Supplement: Supplementary file 9 [file Image_8.jpeg]

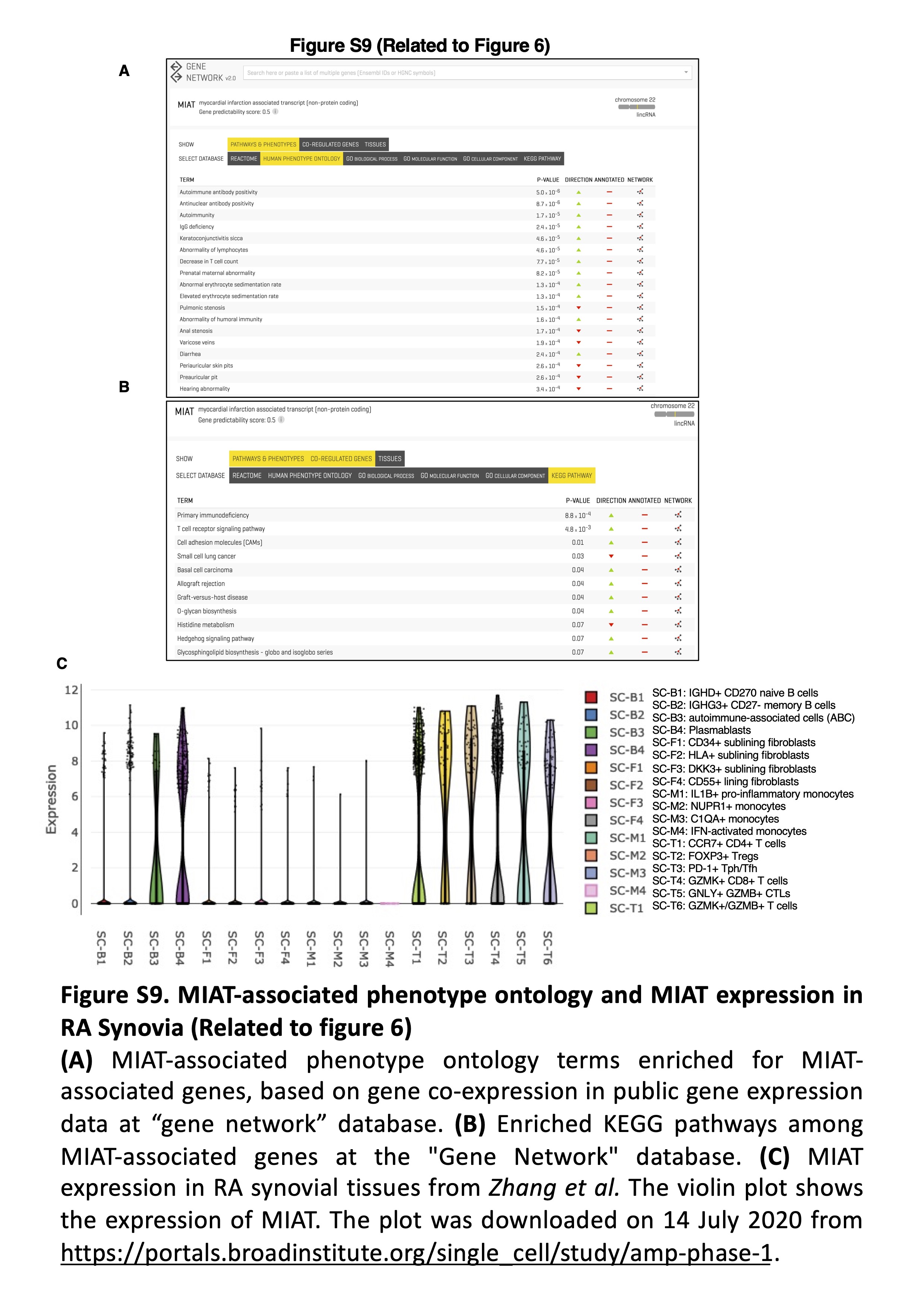

Supplement: Supplementary file 10 [file Image_9.jpeg]

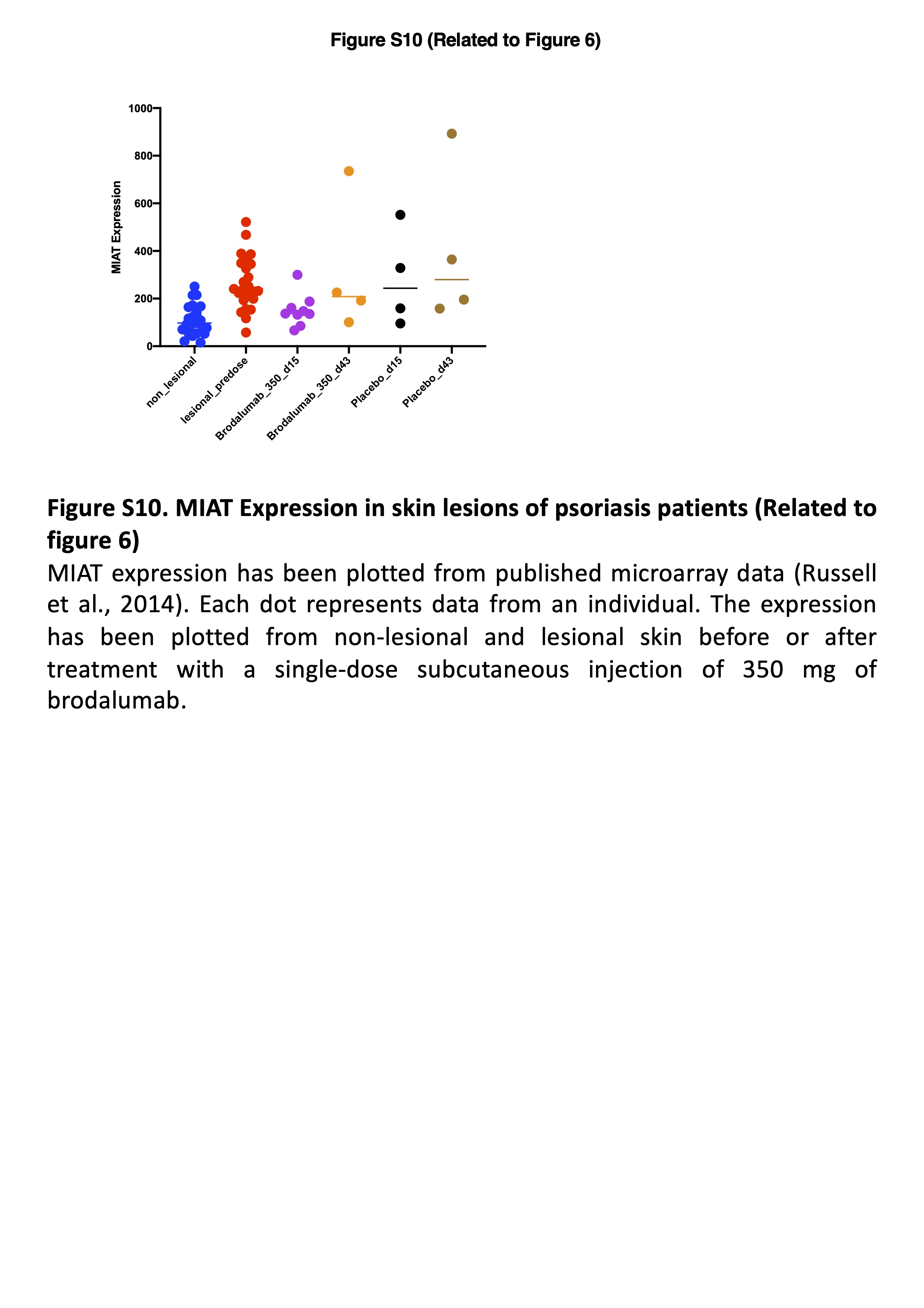

Supplement: Supplementary file 11 [file Image_10.jpeg]
